# Design and Validation of a Periodic Leg Movement Detector

**DOI:** 10.1371/journal.pone.0114565

**Published:** 2014-12-09

**Authors:** Hyatt Moore, Eileen Leary, Seo-Young Lee, Oscar Carrillo, Robin Stubbs, Paul Peppard, Terry Young, Bernard Widrow, Emmanuel Mignot

**Affiliations:** 1 Center for Sleep Sciences and Medicine, Stanford University, Palo Alto, California, United States of America; 2 Department of Electrical Engineering, Stanford University, Palo Alto, California, United States of America; 3 Department of Population Health Sciences, University of Wisconsin-Madison, Madison, Wisconsin, United States of America; Charité - Universitätsmedizin Berlin, Germany

## Abstract

Periodic Limb Movements (PLMs) are episodic, involuntary movements caused by fairly specific muscle contractions that occur during sleep and can be scored during nocturnal polysomnography (NPSG). Because leg movements (LM) may be accompanied by an arousal or sleep fragmentation, a high PLM index (i.e. average number of PLMs per hour) may have an effect on an individual’s overall health and wellbeing. This study presents the design and validation of the Stanford PLM automatic detector (S-PLMAD), a robust, automated leg movement detector to score PLM. NPSG studies from adult participants of the Wisconsin Sleep Cohort (WSC, n = 1,073, 2000–2004) and successive Stanford Sleep Cohort (SSC) patients (n = 760, 1999–2007) undergoing baseline NPSG were used in the design and validation of this study. The scoring algorithm of the S-PLMAD was initially based on the 2007 American Association of Sleep Medicine clinical scoring rules. It was first tested against other published algorithms using manually scored LM in the WSC. Rules were then modified to accommodate baseline noise and electrocardiography interference and to better exclude LM adjacent to respiratory events. The S-PLMAD incorporates adaptive noise cancelling of cardiac interference and noise-floor adjustable detection thresholds, removes LM secondary to sleep disordered breathing within 5 sec of respiratory events, and is robust to transient artifacts. Furthermore, it provides PLM indices for sleep (PLMS) and wake plus periodicity index and other metrics. To validate the final S-PLMAD, experts visually scored 78 studies in normal sleepers and patients with restless legs syndrome, sleep disordered breathing, rapid eye movement sleep behavior disorder, narcolepsy-cataplexy, insomnia, and delayed sleep phase syndrome. PLM indices were highly correlated between expert, visually scored PLMS and automatic scorings (r^2^ = 0.94 in WSC and r^2^ = 0.94 in SSC). In conclusion, The S-PLMAD is a robust and high throughput PLM detector that functions well in controls and sleep disorder patients.

## Introduction

Periodic Limb Movements (PLMs) are episodic, involuntary movements caused by specific muscle contractions that occur during sleep and can be scored during nocturnal polysomnography (PSG). Because leg movements (LM) may be accompanied by an arousal or sleep fragmentation, a high PLM index (e.g. more than 15) may have an effect on an individual’s overall health and wellbeing [1]. Unfortunately, however, little is known about the cause of PLMs or their impact on wellbeing, daytime sleepiness or insomnia symptoms, especially in isolation of Restless Legs Syndrome (RLS). RLS is a movement disorder characterized by sensations and an urge to move the legs in the evening, is often associated with PLMs, with 85–95% of RLS patients having significant PLMs during sleep (PLMS) [2] ^,^ [3] ^,^ [4]. A study review of cardiac risk for RLS and PLMS found associations between PLMS and congestive heart failure [5]. Additionally, patients with RLS were at higher risk for heart disease and hypertension [5]. While the relationship between PLMs and RLS has been thoroughly investigated [6], PLMs also frequently occur without RLS symptoms. PLMs are known to be associated with several other disorders and pathologies such as depression, cardiovascular disease, hypertension, rapid eye movement (REM) behavior disorder, narcolepsy, Parkinson’s disease and multiple system atrophy [7], [8], [9], [10], [11].

PLM scoring rules have evolved over time and are based on the amplitude and duration of the event as well as the time between limb movements. In 1980, Coleman et al. [12] first reported periodic movements in sleep as “repetitive, nonepileptiform movements of one or both lower extremities occurring primarily in non-REM sleep” which recurred “approximately every 30 seconds.” In 1993, the Atlas Task Force, part of the American Sleep Disorders Association (ASDA, now AASM), defined leg movements (LMs) in polysomnograms (PSGs) as increased electromyogram (EMG) activity lasting between 0.5 and 5.0 seconds (sec), in excess of 25% of the recorded voluntary flexion during calibration that should be scored during sleep or wakefulness [13]. LMs are defined as periodic if 4 or more successive movements are observed in sleep, with the possibility of some of these movements continuing across sleep-wake transitions. LMs associated with respiratory events were scored separately, and LMs occurring just after an arousal are excluded. The World Association of Sleep Medicine (WASM) set forth standards for recording and scoring PLM in 2006 (referred to hereafter as *WASM 2006*). WASM 2006 adds considerable detail and description regarding PLM for clinical and research purposes (e.g. additional emphasis on RLS and investigation of morphology and characteristics of LM and PLM). It follows the ASDA’s original PLM arousal criteria with minor modification. Clinical criteria for identifying LM and PLM were condensed in the AASM Manual for Scoring Sleep in 2007 (hereafter referred to as *AASM 2007*). The AASM 2007 and WASM 2006 are very similar in regard to clinical detection of LM and PLM. Both define significant LM (or LM eligible for PLM candidacy) as a 0.5–10 sec period where EMG activity recorded by same configuration from the left or right anterior tibialis (LAT/RAT) exceeds 8 µV above baseline and then falls below 2 µV from baseline for 0.5 sec or longer [14], [15]. The ASDA, WASM 2006, and AASM 2007 all define PLM as the consecutive sequence of four or more LMs whose inter-movement intervals are between 5 and 90 sec [13], [14], [15].

There are subtle differences between the WASM 2006 and AASM 2007. The WASM 2006 provides allowances for baseline noise changes, while the AASM 2007 does not. The WASM 2006 allows PLM to continue from sleep to wake and vice versa, and requires reporting of both PLMs during sleep (PLMS) per hour of sleep (PLMS/h) and PLM during wakefulness (PLMW) per hour of wake (PLMW/h). Wakefulness is defined anytime a subject is awake in bed with their legs horizontal (i.e. not sitting up) and the lights off (to include wake before sleep onset). The AASM 2007 does not explicitly state whether LM series can be validated across intermittent wakefulness, although as this was already defined in the ASDA 1993, it is implied. The WASM 2006 gives additional guidance for handling special LM scenarios (e.g. a middle LM with inter-movement interval less than 5.0 sec from the first LM that breaks the otherwise acceptable first to third LM inter-movement interval, is ignored), which are not found in the AASM 2007. Finally, the WASM 2006 associates LM with respiratory events using a 0.5 sec window about the critical breath following hypopnea/apnea, and removes any found events from PLM inclusion [15]. The AASM 2007 broadens the respiratory exclusion region to exclude all LMs occurring 0.5 sec before until 0.5 sec after a respiratory event [14].

Because PLMs are discrete and well defined events within the nocturnal polysomnography signal, automatic detectors have been created to identify and quantify PLMs during sleep. These detectors were however designed and validated using PSG data generated in relatively small groups of RLS patients versus healthy controls (no sleep disorder) recorded under optimal conditions. In 1996, Tauchmann proposed a PLM detection algorithm where optimal parameters were determined using a training-validation split of 1,671 and 1,740 visually scored LMs (respectively) from five PSGs [16]. Wetter adjusted this algorithm in 2004 and validated it using 8,300 visually scored LMs from PSGs of 10 patients diagnosed with RLS [17]. In 2006, Ferri proposed a detector with parameters optimized using Receiver Operating Characteristics (ROC) curves and validated against visually scored LMs in 15 patients diagnosed with RLS and 15 controls [18]. These detectors did not need to address LMs surrounding respiratory events because sleep disordered breathing (SDB) was an exclusion criterion.

In this study, we report on the building and validation of a new PLM detector, the Stanford PLM automatic detector (S-PLMAD). This detector was constructed using a large epidemiological and clinical sample totaling 1,073 subjects, and validated against manually scored studies (per AASM 2007 criteria) in 78 PSG individuals selected for known sleep disorders and abnormalities. The S-PLMAD reports on PLMS/h and PLMW/h. In all these calculations, PLM sequences are still counted (and corresponding LM in wake or sleep respectively) even if interrupted by intermittent wakefulness as suggested by the WASM 2006. This is the largest sample used to validate a PLM detector, to date, that we are aware of.

## Methods

Human nocturnal PSG recordings were obtained from previous, Institutional Review Board authorized studies. The collected data was further de-identified for analysis and work related to this manuscript submission.

### Cohorts used in the analysis

Nocturnal PSGs from the Wisconsin Sleep Cohort (WSC) [19] and the Stanford Sleep Cohort (SSC) were used in this study. Electroencephalography (EEG), electrooculography (EOG), and chin EMG were used to score sleep stages for each 30 second epoch using standard R&K criteria [20].

#### WSC

Volunteers with PSG files available (n = 1,073) were selected from this cohort. Table 1 presents demographic data on the entire data set and with subjects stratified by an apnea-hypopnea index (AHI)>15. The WSC is a longitudinal study of sleep habits and disorders in the general population. [19] It was established in 1988 from a sample of employees of four state agencies in south central Wisconsin, USA, aged 30–60 years. The first PSGs for each subject that were performed between 2000 and 2004 were used. The studies were exported as EDF files and paired with scoring files of stages and events. The timeframe was selected to be closest to a RLS survey mailing performed in 2003 [21] **.** Sleep was characterized using a 16-channel PSG recording system (16-channel Grass-Telefactor Heritage digital sleep system Model 15). Leg EMG was combined from a pair of leg electrodes placed over the anterior tibialis of each leg. Arterial oxyhemoglobin saturation was measured by pulse oximetry using a 3 sec averaging rate. Oral and nasal airflow were measured using thermocouples (ProTech). Nasal air pressure was measured with a pressure transducer (Validyne, Northridge). Thoracic cage and abdominal respiratory motion were measured with inductance plethysmography (Respitrace, Ambulatory Monitoring). These signals were used to identify SDB events. Apnea was defined as a cessation of airflow lasting ≥10 sec. Hypopnea was defined as a decrease in airflow accompanied by a ≥4% reduction in oxyhemoglobin saturation and is close to the AASM 2007 recommended (Medicare) criteria for scoring hypopneas [22]. AHI was defined as the average number of apneas plus hypopneas per hour of objectively measured sleep. In this cohort, LMs in PSGs were initially defined per ASDA 1993 criteria but using 50% instead of 25% of recorded voluntary flexion during calibration as the threshold. Additionally, the WSC scoring policy counts LMs within 4.0 sec of each other as one LM. LMs associated with respiratory events were variably scored. We considered these manually scored LMs (per modified ASDA 1993 criteria) as a preliminary training set.

**Table 1:** Wisconsin Sleep Cohort (WSC).

	Overall sample			Validation subsample		
	All (1073)	AHI≤15 (738)	AHI>15 (264)	RLS*, AHI≤15 (20)	AHI≤15 (20)	AHI>15 (20)
Demographics						
Age	56±0.24	55.3±0.28	57.8±0.49	54.7±0.67	55.1±0.63	54.5±0.72
Sex, Male (%)	53.2%	48.9%	63.3%	50.0%	50.0%	50.0%
Clinical Data						
BMI (kg/m^2^)	31.7±0.22	29.8±0.22	35.4±0.45	31.1±1.25	31.1±1.25	35±1.43
AHI	12±0.50 (1004)	4.71±0.15	32.2±1.11	5.64±0.96	5.18±0.80	31±3.97
AHI>15 (%)	26.5% (1004)	0.0%	100.0%	0.0%	0.0%	100.0%
PSG						
TST (hour)	6.15±0.03	6.28±0.04	6.08±0.06	6.33±0.23	6.41±0.19	6.21±0.24
WASO (hour)	1.20±0.02	1.10±0.02	1.31±0.04	1.00±0.11	1.07±0.12	1.17±0.15

Data are mean ± Standard Error Mean, or percentage. The number of subject used for calculations are shown in parentheses, when different from total sample. RLS* = Presence of RLS “symptoms”, as described in the material and method section. AHI is the apnea hypopnea index calculated as the number of manually scored respiratory events per hour of sleep. BMI is body mass index; PSG is nocturnal polysomnography. TST is total sleep time; WASO is Wake After Sleep Onset. Patients using Positive Airway Pressure therapies, e.g. CPAP were excluded from AHI categories.

A subset of the WSC data was then re-scored to create a PLMS/h gold standard. To do so, a registered Polysomnographic Technician (EL) scored PLMS according to AASM 2007 criteria with minor modifications based on her professional judgment to create a gold standard to validate the detector from. Table 1 describes this subset of WSC patients. Collectively, the WSC gold standard contains 5,387 PLMs from sixty age and gender-matched participants selected from one of three groups: (1) RLS symptoms without SDB (n = 20); (2) SDB without RLS symptoms (n = 20); (3) neither RLS symptoms nor SDB (n = 20). WSC subjects were identified as having RLS symptoms based on responses to a questionnaire sent to the entire parent cohort in 2003 as described in a previous study [21]. The questionnaire did not address all RLS diagnostic criteria put forth by the National Institutes of Health [23], notably it did not ask for the symptoms to be worse at night. For this reason, we called patients positive for these questions as having “RLS symptoms”**.** Participants with RLS symptoms reported they felt: (a) Repeated urge to move legs and (b) Strange and uncomfortable feelings in the legs weekly or more often plus that these feelings (c) got better when they got up and started walking and (d) disrupted their sleep. SDB was defined based on an AHI cut off of 15 events per hour.

#### SSC

This sample is a naturalistic sample of 760 successive patients (Table 2), including a wide range of sleep disorders, recruited to the Stanford Sleep Disorders Clinic and who had a nocturnal PSG from 1999–2007 [24] **.** Table 2 reports on summary statistics broken down by diagnostic category. The only exclusion criterion was the use of continuous positive airway pressure (CPAP) device for previously documented sleep apnea. PSGs were collected using Sandman Elite digital sleep software and Sandman SD32+ amplifiers. The Stanford Sleep Disorders Clinic protocol exceeds the AASM’s clinical guidelines for the assessment of SDB by recording extra respiratory signals and using additional precision and processing. Eighteen channels of information are recorded including: EEG, EOG, EMG of the submentalis muscle as well as the anterior tibialis muscles of each leg which were also combined into a single leg EMG, electrocardiogram (ECG), snore using neck vibration, breathing effort using Braebon respiratory inductance plethysmography (RIP) system, airflow from Braebon PureFlo Duo Cannula and Nasal Pressure sensors and oxygen saturation (SpO2) through Finger PhotoPlethysmography Pulse Rate. EEG was recorded from conventional 10–20 system electrode sites using a 256 Hz sampling rate. EMG, ECG and snore signals used a sampling rate of 512 Hz while the sampling rates were 64 Hz for breathing effort and airflow and 4 Hz for SpO2, pulse rate. All AC channels used were hardware filtered between 0.1 Hz and 0.45 times the sampling rate and leg EMG channels were additionally high pass filtered at 10 Hz. Apneas were defined as a cessation of airflow lasting ≥10 sec. Hypopneas were defined as a ≥30% reduction in nasal pressure signal excursions and associated ≥4% desaturation or arousal. The hypopnea definition is similar to the alternate AASM 2007 or Chicago criteria.

**Table 2:** Stanford Sleep Cohort (SSC).

	All (760)	Delayed PhaseSyndrome (14)	Insomnia (141)	Narcolepsy (19)	REM BehaviorDisorder (4)	Restless LegsSyndrome (23)	Sleep DisorderedBreathing (607)	Other (39)
Demographics								
Age	45.9±0.52	36±4.67	46.2±1.29	40.2±5.09	59.3±3.84	49.2±3.10	45.5±0.59	42.2±2.62
Sex, Male (%)	58.8%	64.3%	45.4%	42.1%	100.0%	52.2%	58.5%	43.6%
Clinical Data								
BMI (kg/m^2^)	27.1±0.24 (741)	25.7±2.76	25.7±0.39 (140)	26.7±1.08	28.9±0.75	24.9±1.38	27.3±0.27 (599)	27.5±1.37
AHI	13.7±0.70	9.77±2.82	10.9±1.37	10.3±2.80	43±16.83	11.7±3.89	15.5±0.81	10.1±3.01
AHI>15 (%)	33.0%	28.6%	27.0%	21.1%	75.0%	26.1%	36.7%	23.1%
PSG								
TST (hour)	6.12±0.04	6.18±0.34	6.13±0.10	6.69±0.24	6.01±0.66	5.96±0.26	6.14±0.05	5.91±0.22
WASO (hour)	1.33±0.03 (759)	1.10±0.26	1.27±0.07	1.45±0.26	1.38±0.29	1.55±0.23	1.34±0.04 (606)	1.44±0.18 (38)

Data are mean ± Standard Error Mean, or percentage. The number of subject used for calculations are shown in parentheses if different from the total. For abbreviations, see legend to Table 1.

PSGs from 18 subjects were selected from the SSC for a second gold standard validation. A sleep physician (SL) manually scored PLM in sleep, according to AASM 2007 criteria, in this subsample of data, which is enriched with specific sleep pathologies. The SSC gold standard consists of age and gender matched patients having the following diagnoses (i.e. three patients per group): Insomnia, Narcolepsy, REM behavior disorder, RLS, SDB, and other (head trauma with excessive daytime sleepiness, depression, and night terrors).

### Statistical analysis

Data are reported as means ± standard errors of the means (SEM) unless otherwise specified. Two group comparisons are performed using t-tests or χ-squares, whenever most appropriate. Multi-group comparisons were performed using one-way analysis of variance. Statistical significance was set at p<0.05, although results of interest were only discussed when p<0.01 considering the large sample size and multiple testing issues.

Performance for the final S-PLMAD was evaluated first by correlating PLMS/h from individual subjects derived from the detector versus manual scoring (gold standard). This was done using Pearson’s in 60 subjects selected from the WSC and 18 subjects selected from the SSC (see above for subject selection). Bland-Altman diagrams were then used to assess differences between PLMI scorings derived automatically and manually using the same cohort selections.

In addition, we also conducted statistical analysis on the quality of detection at the individual leg movement level using ROC analysis. In these cases, however, sensitivity, specificity, and accuracy measures are not informative because of the skewed distribution of PLM events compared to their absence. Indeed, in continuous data, there is an overwhelming number of true negative segments where no PLM occur, thus it is advantageous to over-detect, as it only penalizes specificity modestly but can raise sensitivity. Positive predictive and negative predictive values as well as Cohen’s Kappa provide more meaningful insight of the detectors performance and agreement to the visually scored gold standards, thus those statistics were reported.

### Design and preliminary testing of the detector

The WSC was the primary dataset used to establish functionality of the detector, while the SSC sample was used as a true validation and to check whether the detector was robust in the presence of sleep disorders. To establish the detector, we first used the AASM 2007 rules for clinical PLMs, which as mentioned above is very similar to the WASM 2006 (version 1). To meet significant LM criteria, EMG activity recorded from the left or right anterior tibialis (LAT/RAT) must exceed 8 µV for 0.5–10 seconds and then fall below 2 µV from baseline for 0.5 sec or longer**.** PLMs are defined by the consecutive sequence of four or more LMs, whose inter-movement intervals are between 5 and 90 sec [14], [15]. AASM 2007 excludes LM occurring anytime between 0.5 sec preceding to 0.5 sec following a respiratory event (e.g. apnea). To test a first iteration of these rules, version 1 was used on the entire WSC dataset, and compared to LM scored according to the modified ASDA 1993 criteria, because these data were directly available for our use (see description of the WSC cohort). Both the automated and manually scored results were examined to identify outliers and potential artifact issues. Along the way, we also compared different iterations of our detector with other established detectors from Tauchmann [16], Wetter [17], and Ferri [18] ( S1–S3 Tables).

### Testing of other detectors

The Stanford’s EDF Viewer (SEV) was used to visualize PSG epochs and LMs detected by manual scoring and by individual detectors. SEV is a PSG processing MATLAB toolbox with a batch that automates power spectral analysis along with classification and detection algorithms across arbitrarily sized datasets [25] The original PLM detection algorithms from Tauchmann [16], Wetter [17], and Ferri [18] were implemented in the SEV as presented in their publications. Care was taken to follow the same pre-filtering steps when possible. For example, differences in sampling rates and electrical standards between countries were accounted for (e.g. 50 Hz interference vs. 60 Hz power line interference). As mentioned earlier, LMs were manually scored as part of the original dataset according to WSC 1995 scoring guidelines [26], a modified version of ASDA 1993 criteria. Because LM definitions of the ASDA differ from those of the WASM 2006 and AASM 2007 definitions, we chose to use these data as a preliminary training sample and, in this case only, adjust the maximum LM duration of all detectors (including ours) to 5.0 sec for fair comparison. ROC curves were drawn against 119,277 originally scored LMs. Sensitivity, specificity, Cohen’s Kappa, positive and negative predictive values, accuracy and total LM count were explored using per leg movement comparisons by subject (S2 Table).

Ferri and Wetter’s detectors looked the most promising in terms of sensitivity, (S2 Table), but these detectors identified almost ten times more LMs than manual scoring. The abnormally high performance is a problematic feature of ROC analysis in cases like this, where a large number true negatives (absence of leg movements detected by manual and automatic scoring) gives a false impression of high performance [27]. In this context, signal detection theory indicates that the positive predictive value (PPV) and Cohen’s Kappa offer more insight into true performance. Using these metrics, our detector outperformed the other algorithms (S2 Table). Examination of the data did not reveal a failure of the other detectors’ methodologies, which were sound, but rather a lack of robustness to noisy EMG signal quality, which they had not been trained on. Indeed, these detectors were typically evaluated and developed using high quality EMG data, with limited noise or artifact, taken from subjects screened for SDB. In addition, we cannot exclude the possibility that in spite of our efforts we were unable to truly replicate the exact algorithms used in previously published detectors.

Even our initial detector’s performance was suboptimal and we next examined outlier studies, studies where PLMS/h scored data differed significantly from automatically detected scores on a correlation plot. Returning to individual studies, the following sources of problems were revealed: (1) ECG contamination in leg movement channels created false detections; (2) variable baseline signal noise affected the 8 µV rule, leading to over and under detection; (3) delayed LMs following sleep disordered breathing events were not omitted by the restrictive 0.5 sec window of the AASM 2007 criteria, while intermediate, respiratory unrelated LM were; (4) fragmentary myoclonus.

### Final Detection Algorithm

**Figure 1:**
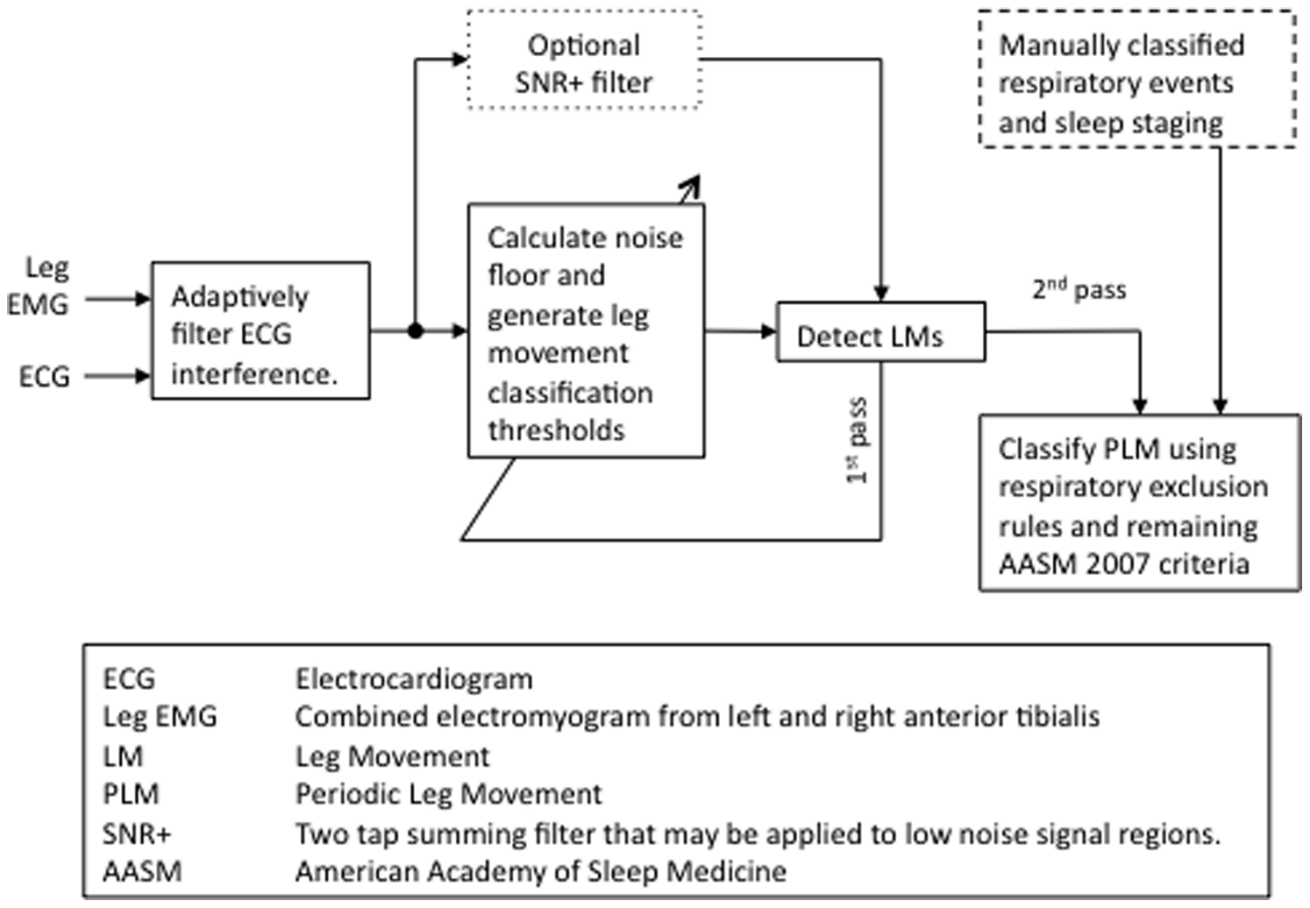
Flow chart of ten-step PLM detection algorithm. First pass LM detections are used to update the noise floor and generate the three amplitude thresholds a second time. The second pass LM detections are subsequently classified or rejected using AASM 2007 PLM scoring criteria, with our modification for respiratory exclusion.

As outlined in Fig. 1, the final PLM detector presented in this manuscript, the S-PLMAD incorporates salient features of previously published detectors and adds several key innovations, particularly in regard to handling noise and artifact. Its design followed a number of iterations not described in sequence for the sake of simplicity. If available, signals from both legs are merged into a single signal as allowed by WASM 2006 and AASM 2007. The final algorithm applies an adaptive filter to the leg EMG to remove ECG interference. A two-pass noise floor calculation is made of the cleaned signal to establish dynamic upper, lower, and falloff amplitude detection thresholds for each sample point. An optional 2-tap summing filter may be applied to the adaptively cleaned signal in places where the noise measurement falls below 2 µV; referred to as the *SNR+* option. The 0.15 sec root mean square (RMS) value of the adaptively cleaned signal is compared to these amplitude thresholds and a candidate LM is registered from the point the RMS first exceeds the upper threshold until the last point it is above the falloff amplitude before subsequently falling below the lower threshold for 0.05 sec (adjusting Ferri’s dual threshold approach). Candidate classifications within 0.1 sec are next bridged (similar to Tauchmann), and candidate LMs subsequently less than 0.75 sec, rejected (i.e. 0.5 sec AASM criteria plus 0.25 sec to account for signal stretching caused by the filters). Remaining LMs within 2.0 sec are then merged a final time as done by Wetter [17], and candidate LMs greater than 10.0 sec are rejected. Merging within 2.0 sec is helpful in grouping alternating LMs recorded to a single channel.

The area under the curve (AUC), calculated by trapezoidal integration, is taken from cleaned data over the range of points corresponding to each candidate LM. If the candidate LM’s AUC is greater than half the upper amplitude threshold it is retained as a LM, otherwise it is rejected. A final respiratory exclusion window is applied such that detected LMs that fall between 5.0 sec before until 0.5 sec following a manually scored respiratory event or from 0.5 sec before until 5.0 sec following its offset, are removed while any LMs during respiratory events which do not fall in these boundary exclusions are not. Remaining LMs are automatically reviewed by the final detection algorithm to determine whether they meet criteria for a string of PLMs according to AASM 2007’s inter-movement interval and frequency requirements (i.e. four or more consecutive LM with inter-movement interval between 5 and 90 sec). Explanation of rationale following these design decisions follows, and a more extensive flow diagram of these steps can be found in S1 Figure.

### Adaptive noise cancelling to remove ECG artifacts from leg EMG

Upon inspection, we found that interfering cardiac signal activity contaminated some studies and resulted in a large number of false positive detections of LMs. A low pass filter was initially used to remove this interference, however true muscle activity was indiscriminately attenuated which led to increased false negative detections. An adaptive recursive least squares filter, described by He et al. for filtering EOG artifact from EEG signals [28], was deployed with great success.

To address possible cases where the ECG signal may be missing (e.g. some ambulatory configurations), we created a time-advanced copy of the LAT/RAT channel as the desired reference channel response as suggested by Widrow et al. [29]. A small filter size and time advance allows the filter to adapt to the highly periodic cardiac interference and not LM. We found a five weight adaptive filter with a five-sample, duplicate source-channel, reference signal advance to effectively handle cardiac interference in a solitary input channel configuration. Although this second method was effective, it was less optimal and was not used in this particular dataset where ECG is always available.

Description of the filter and examples of its application in removing cardiac interference in the LAT/RAT channel without attenuating true leg muscle activity, as observed with conventional finite-impulse response filtering, are provided in S2 and S3 Figures.

### Variable amplitude thresholding to address variable or excessive baseline noise

Typically, the noise floor is measured during the preflexion calibration step in PSG studies, and the LM detection thresholds are adjusted accordingly (i.e. 8 µV above baseline followed by a drop below the 2 µV baseline offset). Unfortunately, establishing a constant offset of 8 µV for the entire study (e.g. AASM 2007) is insufficient in separating true signal from noise as the noise floor increases. As the baseline noise floor begins to increase, sporadic alterations in amplitude become common along with false detections from the frequent transitions between the upper and lower thresholds.

Patient movement or high electrode impedance levels deter automated detectors that rely on a statically calculated baseline noise level. In these cases, the mean baseline signal may be high, for example 6 µV, and a very small increase may be sufficient to trigger a false LM detection. The WASM 2006 recommends increasing the baseline in areas where EMG activity is raised due to prolonged muscle flexion – a related problem. The new baseline is taken from the average EMG amplitude during this time [15]. This recommendation gives flexibility to visual scoring, but is not well suited for automated processing nor is it helpful when calibration is missing or not performed (e.g. ambulatory studies).

To address this issue, we developed and used a variable amplitude thresholding (VAT) algorithm to handle changes to baseline noise. VAT uses a mathematical model of the noise floor from two passes of the LAT/RAT EMG channel in order to establish optimal thresholds for PLM detection. A first-pass measure of noise is taken as the 20 sec moving average filter of the data. An upper and lower threshold are calculated from this noise floor, and sections of data deemed LM on the first pass are then set to a lower value and a second noise floor measure is made using the adjusted data and additional LMs classified using the upper and lower thresholds calculated from the second pass noise floor measure. Thus, the amplitude criteria can now follow or *ride* along baseline noise and be flexible to changes as they occur through the study. Further noise floor elevation effectively shuts the detector off by raising the signal to the point where it either no longer falls below the lower threshold or does but will have exceeded the maximum LM duration (i.e. 10 sec).

We developed the following system to determine the noise floor and corresponding upper and lower threshold for optimal LM detection. The noise floor, *η*, is defined for sample n as.
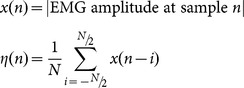
where x(n) is the EMG voltage measured at sample n. We choose *N* so that the noise floor is evaluated from the 20 sec surrounding area of each sample and covers twice the maximum LM duration allowed. Letting *U* be the original upper threshold scalar value (i.e. 8 µV) and *L* the lower threshold scalar (i.e. 2 µV), the PLM detection thresholds for each sample are defined as



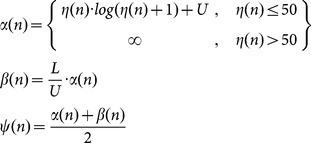
where *α*(*n*), *β*(*n*), and *ψ*(*n*) correspond to the upper, lower, and offset thresholds at sample index *n*. Log represents the natural logarithmic function.

The EMG RMS, *y(n)* is defined using a 0.15 sec window with sampling rate *f_s_* as
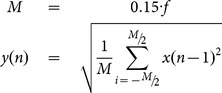



This is compared to the three thresholds to determine candidate LM onsets and offsets. LM onset is defined when *y(n)* first exceeds *α*(*n*). LM offset is defined as the last point *y(n)* exceeds/after onset and before subsequently falling below *β*(*n*_1_+*k*) for 0.05 sec. Detections separated by 0.1 sec or less are merged.

A problem with a single pass noise floor calculation is that the contribution from true LMs are not taken into account. True LMs averaged into the noise floor may elevate the detection thresholds to the extent that nearby LMs are missed. Using a second pass, portions of the initial input signal *x(n)* containing first pass LM are replaced with half the lower threshold for that same region (i.e. *β*(*n*)/2). The noise floor and corresponding thresholds are then calculated again before the second, final detection pass is made. Additional passes can be defined in the same manner, however doing so may overly reduce the noise floor and lead to false positive detections. Similarly, setting the first pass signal sections with detected LMs to zero, instead of *β*(*n*)/2), also overly reduces the noise floor and results in false positive detections.

Alternatively, EMG signal strength may be attenuated at times from weak recording or physiological generation and can be corrected for using a signal-to-noise ratio enhancement (i.e. *SNR+*) option. We found that in some cases, very low noise at baseline allows the clear visualization of LMs that are not captured by our detector because they are smaller than 8 µV. This was a particularly common occurrence in the WSC Gold Standard where the technician annotated PLM based more on visible periodicity seen on one and two minute time scales than quantitative measures of change from baseline.

The SNR+ option increases signal strength when the noise floor falls below 2 µV by application of a two-tap finite impulse response (FIR) summing filter which increases signal strength from 0 to 33 Hz while attenuating its strength for frequencies above 33 Hz (i.e. high frequency activity commonly associated with noise). S4 and S5 Figures describe the filter in detail and include motivating examples. The rationale for providing the detector both with and without the SNR+ option is because the SNR+ is not in strict accordance to the 8 µV criteria of the AASM 2007, although as it will be outlined below, it correlated better with technician trained scoring of PLM.

### Fragmentary or transient myoclonus and spurious noise

Fragmentary myoclonus, brief (<200 msec) spikes of EMG activity [13], and other spurious noise are inadvertently *smeared* by the moving average filter applied to smooth the data, and produce false LM. Smearing effects are minimized by the short 0.15 sec RMS filter in combination with our 0.05 sec fall time and merging criteria.

The 0.05 sec fall time used to identify initial LM offset allows the detector to isolate short spikes in EMG activity and quickly *reset* to make new detections. Longer fall times leave the detector *on*, prolonging the opportunity to find movement, and making it susceptible to lingering, fragmentary activity. For example, a slowly declining LM followed by spurious noise becomes artificially long; possibly to the point of rejection. The subsequently applied 0.1 sec merge-window handles cases where the short fall time can finely split a true LM in parts, and falls in line with Tauchmann’s approach. [16].

Despite these tactics, fragmentary myoclonus or other sharp, short duration spikes in leg EMG activity may still raise the RMS signal to detection levels. As a final test against false detection, we calculate AUC of the original input signal’s magnitude at each candidate LM (i.e. onset to offset) and compare it to the upper detection threshold, *α*(*n*). LMs are relatively stable. They do not quickly rise above and then fall below the detection thresholds, but remain near or above it as characterized by the movement. The raw signal’s AUC is used as measure of this stability in comparison to the detection threshold across the detected region. Candidate LMs with AUC less than half the upper noise threshold are rejected from PLM inclusion. Fragmentary myoclonus can be identified, instead of PLMs, by choosing to instead keep candidate LMs with AUC less than this value.

### Respiratory events and artifact

Respiratory events, routinely annotated in nocturnal PSGs, include hypopneas (with 4% desaturation) and apneas (central, obstructive or mixed). The AASM 2007 Scoring Manual places a window from 0.5 sec prior *until* 0.5 sec after respiratory events in which LM activity is excluded from PLM criteria. Using this rule, we found PLMS/h to be greater in individuals with AHI>15, and lesser in those with AHI ≤15. This seemed incongruent with the intention of excluding false LMs from PLM criteria due to respiratory events. Assuming PLMs and SDB are not strongly correlated at the epidemiological level, removing leg movements secondary to SDB should lead to similar PLMS/h values in subjects with and without sleep apnea. The AASM 2007’s 0.5 sec exclusion rule does not meet its expressed intent of eliminating respiratory related LMs from PLM inclusion (S4 Table).

We thought this requirement was overly conservative at the respiratory event boundaries where the 0.5 sec window is too small to catch the link between breathing event and leg movement. Therefore, we investigated the interaction by comparing manually scored respiratory events in the WSC studies (time of onset and offset of hypopneas with 4% desaturation and apneas) to leg EMG activity during *uninterrupted* sleep (i.e. activity surrounding respiratory events ending in or immediately followed by wake were not considered). Leg activity events surrounding respiratory events that were shorter than 15 sec or were within 30 sec of another respiratory event were also rejected from analysis. Indeed, a leg movement preceding a respiratory event may be due to the nearby, previous respiratory event offset, and vice versa.

**Figure 2:**
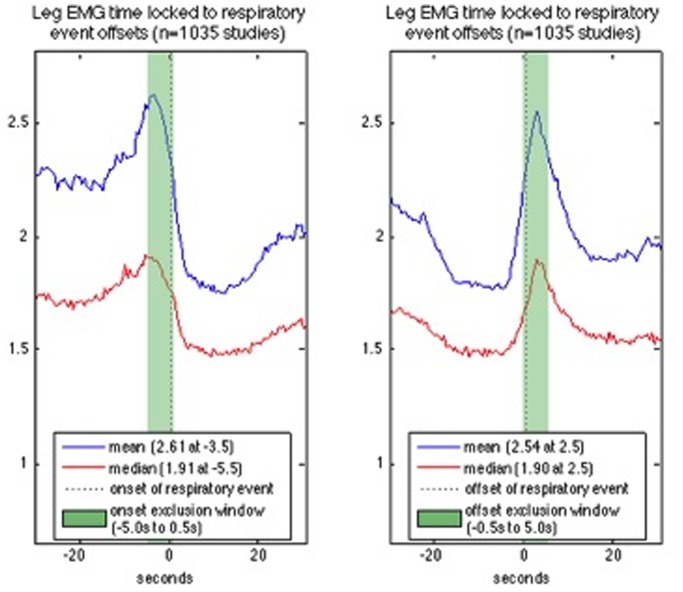
Leg EMG activity time locked to manually scored WSC respiratory events. Expected mean and median absolute leg EMG voltage is calculated in 0.5 s increments starting 30 s prior until 30 s after respiratory event onset (left figure) and each respiratory event offset (right figure). Respiratory events followed by wake, less than 15 s in duration, or which fall within 30 s of another event are removed. Leg EMG activity increases and then decreases prior to respiratory onset with a peak 4.5 s prior to onset, and later decreases before and increases after respiratory offset with a mean peak 2.5 s and median peak 3.5 s.

Fig. 2 shows the expected mean and median leg EMG activity, calculated on 0.5 sec increments time locked 30 sec prior until 30 sec following manually scored respiratory event onset and offset. Leg EMG activity increases and then decreases prior to respiratory onset with a peak 4.5 sec prior to onset, and later decreases before and increases after respiratory offset with a mean peak 2.5 sec and median peak 3.5 sec. The effect of various respiratory exclusion windows on resulting LM and PLM metrics were evaluated using our initial detector with adaptive filtering and a new respiratory exclusion window was selected.

The window to exclude LMs due to respiratory events extends 5.0 sec prior to respiratory event onset until 0.5 sec after onset, and then again from 0.5 sec before to 5.0 sec after the respiratory event’s offset. LMs occurring during the respiratory event that do not fall in the exclusion window may be considered for PLM, which is a departure from the AASM 2007 scoring guidelines.

Examination of respiratory events isolated by 60 sec reveals similar findings, as do examinations of time locked EMG activity in patients with fewer than twenty respiratory events. Additional considerations and details of this analysis are provided as supplementary material (S6–S9 Figures, and S4 Table).

### PLM distribution and characteristics

Our automated detector provides flexibility in terms of reporting PLM indices as a function of sleep stage, sleep (PLMS/h), or wake (PLMW/h). Duration, and specific onsets and offsets of LM and PLM are directly available and allow a variety of further research investigation as recommended in the WASM 2006. This can be particularly helpful when PLM measures need to be tailored for a particular project or question (e.g. follow-up investigation of the mean duration of individual PLMs in RLS [30]). It however does not report on PLMs associated with arousals. Automatic detection of arousal was considered beyond the scope of this first study.

Beside PLM indices and total PLM counts, we also reported LM counts (not necessarily periodic but after removal of any secondary to respiratory events). The Periodicity Index, known to differentiate RLS versus non RLS cases and first introduced by Ferri et al. [2], [31], was also reported as is PLM and LM night ratio, reflecting the ratio of leg movements during the first part versus the second part of the night. Ratios were not calculated for individuals with zero event occurrences in either part of the night. These measures follow previous observations on the circadian effect and clustering of PLM. [32], [33], [34].

Another advantage of computer-based methods is the flexibility with which it is possible to investigate events after those have been detected. For example, cardiac accelerations preceding PLM have been reported consistently with manual, or manually assisted detection methods of PLM [35], [36], [37]. For each event detected as a PLM in a given subject, we computed cardiac activation measures in the ECG surrounding PLM using the method presented by Winkelmann [36]. Heart rate (HR) for the ten cycles prior to and following each PLM is calculated using the R–R interval measured from the ECG at these points and using the heart rate directly before PLM onset as a baseline. That is, the heart rate calculated for the cycle just prior to PLM onset is subtracted from all twenty instantaneous HR calculations made for the PLM. We report two PLM associated cardiac measures: (1) *heart rate delta* which is the difference between the largest normalized HR of the ten cycles HR following PLM onset less the smallest normalized HR in the ten cycles preceding PLM onset, and (2) *heart rate slope* which is the heart delta divided by the number of cycles between the minimum and maximum detected HR values.

## Results

### Validation of the S-PLMAD by independent scorers

In Table 3, individual PLMS/h values derived from AASM 2007 manual scoring (gold standard) are correlated with individual PLMS/h values computed using our detector (with and without SNR+). Table 3 also compares correlations of other previously published detectors. The tables give squared correlation coefficients (r^2^) across groupings of 20 subjects with and without RLS symptoms and SDB within the WSC, and overall for all 18 subjects selected from the SSC.

**Table 3:** PLMS/h comparisons between automatic methods and manually scoring.

	Wisconsin Sleep Cohort				Stanford Sleep Cohort
	RLS*, AHI≤15 (20)	AHI≤15 (20)	AHI>15 (20)	All (n = 60)	All (n = 18)
Tauchmann^b^	0.64	0.32	0.54	0.53	0.49
Wetter^c^	0.12	0.82	0.24	0.25	0.15
Ferri^d^	0.93	0.72	0.85	0.88	0.41
PLM calculator	0.93	0.73	0.84	0.89	0.93
PLM calculator (SNR+)	0.95	0.85	0.94	0.94	0.94

The squared correlation coefficient (r^2^) between PLMS/h determined automatically versus manually is shown in the table for previously published detectors and our PLM calculator with and without the SNR+ option. PLM are classified according to AASM 2007 scoring criteria^a^ with adjustment to LM classification for our classifier as described in the text.

*RLS symptoms as defined in the text.

a Iber C A-IS, Chesson A, Quan SF. The AASM Manual for the Scoring of Sleep and Associated Events: Rules, Terminology and Technical Specifications. Westchester, Ill: American Academy of Sleep Medicine, 2007.

b Tauchmann NPT. Automatic Detection of Periodic Leg Movements. J Sleep Res 1996; 5(4): 273–5.

c Wetter TC, Dirlich G, Streit J, Trenkwalder C, Schuld A, Pollmacher T. An automatic method for scoring leg movements in polygraphic sleep recordings and its validity in comparison to visual scoring. Sleep 2004; 27(2): 324–8.

d Ferri R, Zucconi M, Manconi M, et al. Computer-assisted detection of nocturnal leg motor activity in patients with restless legs syndrome and periodic leg movements during sleep. Sleep 2005; 28(8): 998–1004.

**Figure 3:**
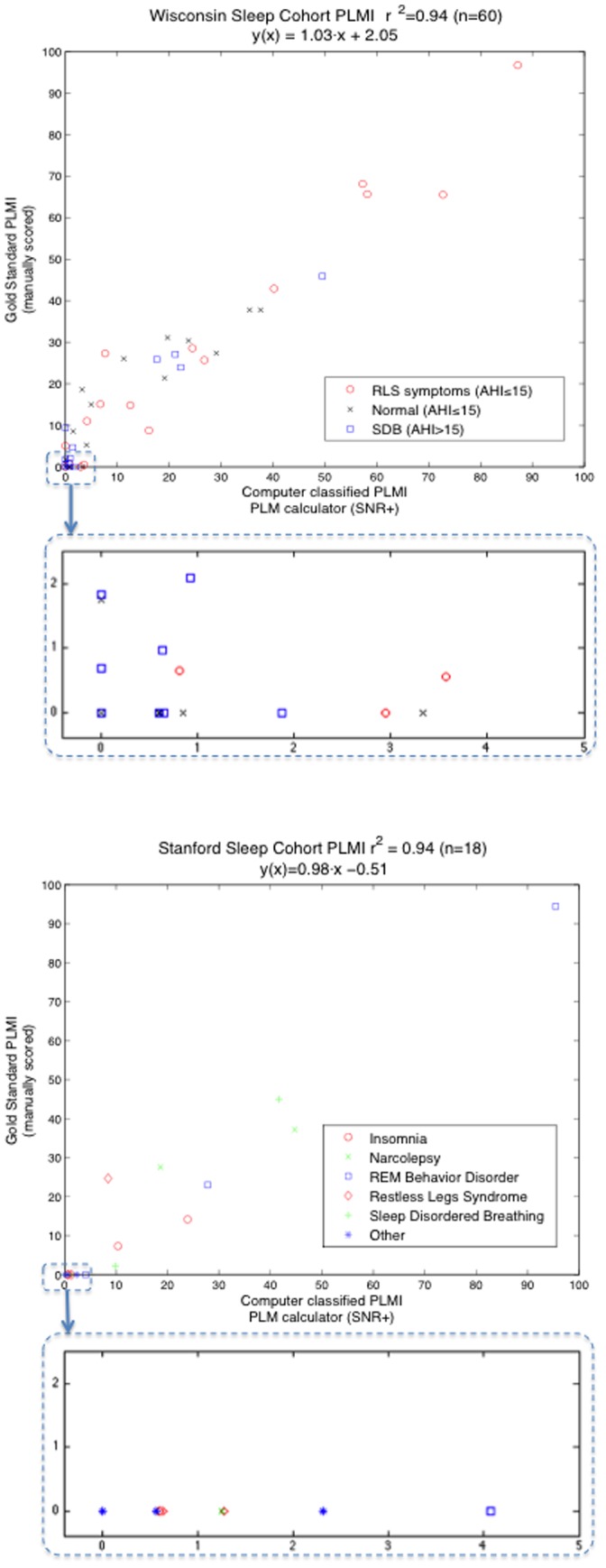
Gold standard PLMI evalution. Comparison of PLMI derived from manual scoring (y-axis) and our automatic PLM calculator using SNR+ (x-axis) in the Wisconsin Sleep Cohort (upper figure, n = 60) and from the Stanford Sleep Clinic (lower figure, n = 18). Below each figure is a zoomed-in view of PLMI scores less than 5.

As can be seen, the S-PLMAD had the best correlations overall and in individual subgroups, with a slight improvement when the SNR+ step was included in the detector. Overall correlation in Fig. 3A and 3B clearly shows the detector functioning well in all groups. The correlation seen in Fig. 3B (SSC sample) is important as diagnoses were enriched for rare diagnosis that we felt could be problematic such as narcolepsy, hypersomnia, REM behavior disorder, obstructive sleep apnea, delayed sleep phase syndrome and insomnia.

**Figure 4:**
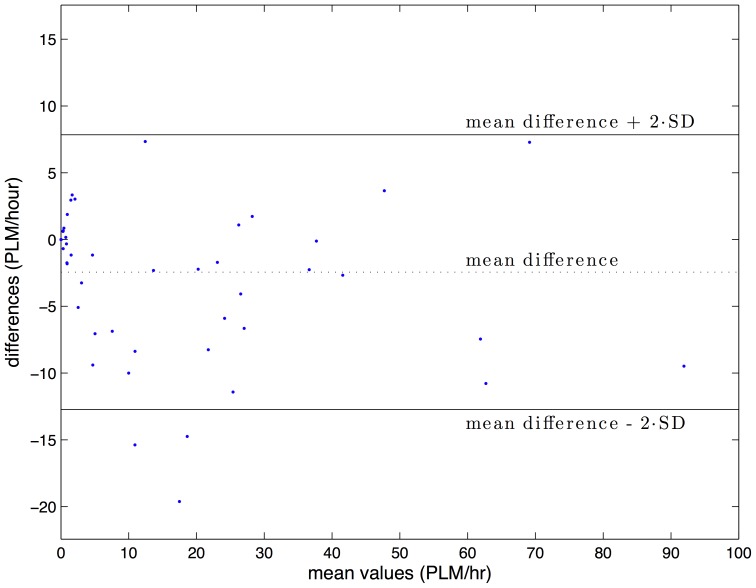
Bland-Altman diagram of PLMI average scoring vs difference of scoring between S-PLMAD and visual detection in the Wisconsin Sleep Cohort (n = 60).

**Figure 5:**
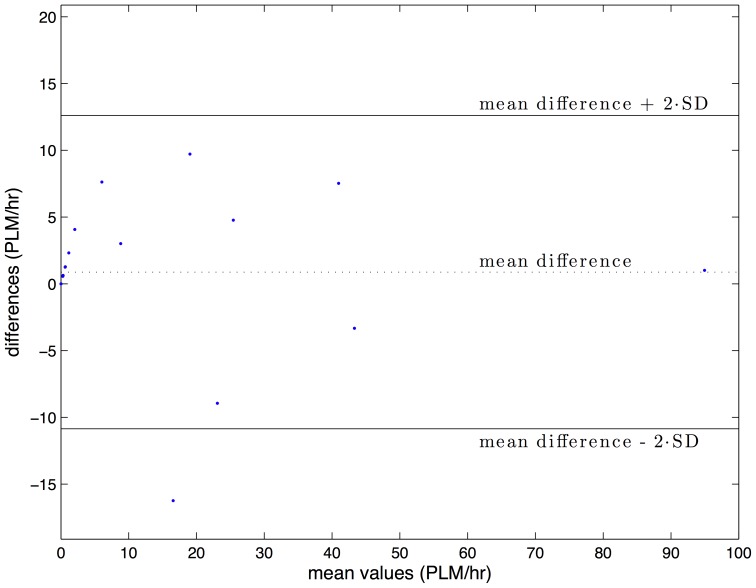
Bland-Altman diagram of PLMI average scoring vs difference of scoring between S-PLMAD and visual detection in the Stanford Sleep Cohort (n = 18).

Bland-Altman diagrams of S-PLMAD and visually scored PLMI in the WSC and SSC validation samples are shown in Figs. 4 and 5. Outliers in both cohorts fall below the 95% difference threshold between S-PLMAD and visual scorings and indicate the S-PLMAD has comparatively underscored PLMI in these subjects. Reexamination of these studies with the scoring technicians revealed physiological PLM activity that was caught visually, but which did not exceed the 8 µV LM threshold required by the S-PLMAD even with the SNR+ option enabled. For this reason, we felt that the detector performed normally and that the manual scoring had not followed recommended guidelines.

S3 Table shows sensitivity, specificity, positive and negative predictive values, Cohen’s Kappa, and accuracy values for discrete PLM scored visually and scored automatically in each cohort. As mentioned in the introduction, these measures are however heavily influenced by the skewed distribution of non-PLM events compared to PLM events that make interpreting specificity, negative predictive value, and accuracy difficult.

### PLM characteristics in the WSC and SSC cohorts

Tables 4 and 5 show summary statistics for PLM metrics in the WSC and SSC. PLMW is shown first followed by PLMS and the periodicity index and other PLM measures calculated during sleep only. As expected, PLM ratio (beginning versus end of night) was high, ranging from a two to four fold increase in both cohorts in contrast to a ratio closer to one computed for all leg movements. This reflects the well known circadian distribution of PLMs, also validating the detector [34].

**Table 4:** Automatically obtained PLM biomarkers in Wisconsin Sleep Cohort.

	AHI≤15 (738)	AHI>15 (264)	All (1072)	AHI≤15 vs AHI>15
PLM/h				
Wake	29.8±1.0	36.6±1.8	31.5±0.8	p = 0.001
Sleep	10.7±0.7	9.62±0.90	10.9±0.6	p = 0.345
Periodicity				
Wake	0.16±0.00 (672)	0.17±0.01 (246)	0.17±0.00 (984)	p = 0.358
Sleep	0.31±0.01 (509)	0.24±0.02 (203)	0.29±0.01 (762)	p<0.001
Count				
LM	152±5	166±7	159±4	p = 0.134
PLM	65.7±4.3	57.2±5.46	64.3±3.4	p = 0.218
Hours evaluated	6.28±0.04	6.08±0.06	6.15±0.03	p = 0.004
Heart rate				
Delta	33.1±0.9 (509)	28.6±1.26 (203)	31.6±0.71 (762)	p = 0.004
Slope	3.37±0.10 (509)	2.90±0.14 (203)	3.21±0.08 (762)	p = 0.006
Night ratio				
PLM	4.82±0.66 (379)	4.39±0.65 (153)	4.62±0.47 (574)	p = 0.638
LM	1.23±0.16 (735)	1.09±0.06 (262)	1.18±0.11 (1065)	p = 0.416

PLM metrics are calculated from PLM classified using our detector (SNR+). PLM/h and periodicity indices are calculated for wake (PLMW/h) and sleep (PLMS/h). Patients not using CPAP and having more two hours of evaluation were grouped according to their apnea-hypopnea index (AHI). CPAP users are not excluded from the “All” category. An hour of the night effect is seen with PLM, occurring almost five times as frequently in the first half of the night compared to the second half (PLM night ratio), while leg movement activity occurs equally across the night in patients with respiratory difficulty during sleep. Note that the periodicity index is artificially affected by excluding intermittent wake and produces a statistically significant higher value in patients with AHI≤15 when examining sleep only.

Data are mean ± Standard Error Mean, or percentage. The number of subject used for calculations are shown in parentheses. Periodicity is the periodicity index. Count is the total number of individual PLM or LM counted per study. Night ratio is the ratio of events classified in the first half of each study divided by the number of events classified in the second half. Heart rate is the normalized cardiac change (beats per minute) time locked to PLM as described in the text. P-values are calculated from the student t-test with significance level of 0.05. Periodicity index, heart rate, and PLM ratio is only calculated in the presence of PLM. Night ratios are only calculated in cases where PLM or LM occur during both the first and second half of the study.

**Table 5:** Automatically obtained PLM biomarkers in Stanford Sleep Cohort.

	All (757)	Delayed PhaseSyndrome (14)	Insomnia(140)	Narcolepsy(17)	REMBehaviorDisorder (4)	RestlessLegsSyndrome (24)	SleepDisorderedBreathing (597)	Other (38)	p
PLM/h									
Wake	32.1±1.0 (752)	36.6±6.8	29.9±2.2	45.6±11.6	62.6±20.6	40.2±6.2	32.0±1.1 (596)	27.5±3.4 (37)	0.076
Sleep	8.59±0.65	5.45±1.91	6.92±1.28	13.1±4.3	32.8±21.4	11.4±4.3	8.23±0.71	6.61±2.65	0.112
Periodicity									
Wake	0.19±0.01 (690)	0.23±0.03 (12)	0.19±0.01 (129)	0.22±0.04 (16)	0.21±0.11	0.22±0.02 (23)	0.19±0.01 (549)	0.22±0.03 (34)	0.684
Sleep	0.23±0.01 (499)	0.23±0.05 (9)	0.21±0.02 (87)	0.26±0.05 (13)	0.34±0.15	0.19±0.04 (21)	0.22±0.01 (390)	0.19±0.04 (23)	0.884
Count									
LM	156±5	129±20	129±9	218±48	350±131	201±31	154±6	139±18	0.006
PLM	50.8±3.8	33.9±11.6	38.7±6.5	82.9±27.1	189±128	59.2±20.8	49.0±4.2	36.5±14.3	0.095
Hours evaluated	6.13±0.04	6.18±0.34	6.12±0.10	6.71±0.27	6.01±0.66	5.97±0.25	6.12±0.05	5.86±0.22	0.446
Heart Rate									
Delta	24.9±0.8 (499)	17.0±2.4 (9)	24.9±2.6 (87)	21.4±2.3 (13)	9.89±1.74	33.1±6.9 (21)	24.3±0.9 (390)	27.4±2.6 (23)	0.259
Slope	2.54±0.09 (499)	1.53±0.20 (9)	2.54±0.29 (87)	2.34±0.26 (13)	0.97±0.15	3.50±0.78 (21)	2.47±0.11 (390)	2.74±0.28 (23)	0.260
Night ratio									
PLM	1.98±0.26 (311)	1.07±0.38 (5)	2.75±1.20 (49)	0.34±0.09 (12)	0.47±0.30	1.75±0.92 (13)	1.97±0.30 (253)	1.60±0.63 (14)	0.868
LM	0.66±0.03 (700)	0.96±0.29 (13)	0.50±0.05 (125)	0.38±0.08 (16)	0.67±0.23	0.45±0.09 (21)	0.66±0.03 (556)	0.46±0.08 (31)	0.158

PLM metrics are calculated from PLM classified using our detector (SNR+). PLM/h and periodicity indices are calculated for wake (PLMW/h) and sleep (PLMS/h). Patients having more two hours of evaluation were grouped according to sleep pathology as determined by formal medical diagnosis. The circadian effect is reversed in narcolepsy, with PLM more likely to occur during the second half of the sleep study, and less extreme in insomnia where PLM are only slightly more frequent (i.e. 33%) in the first half of the study.

Data are mean ± Standard Error Mean, or percentage. The number of subject used for calculations are shown in parentheses. Probabilities (p) are calculated using one-way analysis of variance between groups with a 0.05 significance level. See Fig. 4 for description of terms.

Comparing Table 4 and Table 5 and the two samples overall, PLMS/h were higher in WSC versus SSC, likely reflecting the increased age of the WSC. Other differences appear (notably in PLM night ratio, a reflection of diurnal or circadian control of PLM) and could be explained by age or the nature of the cohort, population-based sample versus clinical sample. Further analysis, outside of the scope of this manuscript may reveal the source for these differences, which overall were relatively modest.

A key feature of our detector in comparison to others is removal of LM in association with SDB. The algorithm, was successful in doing this, as evidenced by PLMS that were not statistically different in subjects with AHI≤15 versus>15 in the WSC or between patients with and without SDB in the SSC. PLMW/h was however significantly higher in subjects with AHI>15 versus ≤15, a difference that could reflect unscored breathing events during wakefulness or other factors (see discussion). We also found that the periodicity index was higher in subjects with SDB, and that heart rate activation following a PLM was lower in subjects with SDB (Table 4).

Another difference was in the HR slope and delta, which were significantly smaller in patients with SDB in the WSC. A sub analysis of these after correction for patients taking beta-blockers suggested this was not secondary to treatment effects of associated high blood pressure (data not shown).

We also computed PLM per sleep stages, and found increased occurrence of PLM during Stage 2 sleep (data not shown). This observation follows that of Ferri et al., who used an automatic detection followed by human confirmation and adjustment [35]. As mentioned above, we also report PLMS and PLMW, and found these measures to be only marginally correlated (r^2^ = 0.17 and 0.26 in SSC and WSC respectively, p<0.001). PLMW has been suggested to be an interesting measure of RLS severity, something that was not explored here.

### PLM by diagnostic groups

Table 5 reports on PLMs across diagnostic groups at the Stanford Sleep Disorders Clinic. As expected, PLMW and PLMS were highest in narcolepsy and RLS; these pathologies are known to have the highest association with PLMs. Data from REM behavior disorder subjects also showed high PLMS, but variance was high in only 4 subjects. Insomnia, delayed sleep phase syndrome and sleep disordered breathing samples had similar PLMS values. Overall the data was in line with what was expected, although ANOVA statistics across groups did not reveal a significant difference. This was likely because the diagnostic groups of interest with expected high PLMS (narcolepsy, RLS) were small in size.

### Code for the Stanford PLM detector

The detector uses the SEV [25]. Source code for both the SEV and the S-PLMAD are available online at either http://www.github.com/informaton or http://www.stanford.edu/~hyatt4 and can be freely modified or integrated into other applications by the open source community.

## Discussion

In this report, we describe a novel PLM detector, the S-PLMAD, and its validation in two independent adult samples (patients-based and population-based cohorts). Our goal was to create a robust detector that would closely approximate manual scoring by experienced technicians using AASM 2007 PLM scoring rules. The detector was optimized to remove false signals from leg channels, such as ECG contamination, or fragmentary myoclonus like patterns. It was also designed to remove LM associated with SDB that could appear periodic. It may use one or two leg channels, although in our cases, two combined leg channels were used in both cohorts examined. We have since extended our investigation to LM and PLM detected from separate leg channels using the S-PLMAD in more recent cohorts. Furthermore, we recently used the S-PLMAD to identify genetic associations of PLM and loci previously identified with RLS [38]. This discovery provides further, indirect, validation of the S-PLMAD.

Our detector was found to be robust, functioning in two distinct samples, a population based sample and a clinical sample, and showed high correlation with expert visual scoring. It is capable of reporting PLM measures across sleep, and, as suggested by WASM 2006 for wake only (a measure suggested to correlate with RLS severity in patients with the disorder). Rules can easily be modified to report PLM indices per sleep stages separately. Unfortunately, however, arousal scoring was not attempted following the detection of individual LM, so that PLMA indices (PLM indices with subsequent arousal) are not reported. We also validate the detector in comparison to human scoring only for PLMS and not PLMW. Future development in this direction may be attempted, but could be difficult considering the polymorphic presentation of arousals in PSG signals across various sleep stages.

Although we started with the AASM 2007 criteria, two modifications of the rules were necessary to improve detection. First, we found that defining a LM as a having EMG signal exceeding 8 µV above baseline and then falling below 2 µV from baseline was difficult to use if background noise was either very high or very low. In the former case, leg movements may still be detectable above baseline, but if starting from a high baseline, may be scored spuriously when the total signal crossed the 8 µV threshold even following a small rise in signal or an artifact. In the later cases, small leg movements may be obvious and periodic, but do not reach the 8 µV threshold. To accommodate these cases, we created the SNR+ option and continually account for changes to the noise floor as it varies from almost none to high (for example 6 µV, see S10 Figure).

Second, we found it necessary to modify exclusion rules for movements surrounding SDB. Exclusion of LM secondary to SDB or how to address SDB in association with RLS is the object of debate, but has not yet reached clear consensus [39]. At one end of the spectrum, some authors have argued that almost all PLM events are secondary or connected to SDB, notably after start of positive pressure therapy of SDB [40] **.** At the other end of the spectrum, some investigators in the RLS field do not score SDB and remove LM events that could be secondary to SDB and associated arousal. To address this issue, WASM 2006 guidelines exclude LM that overlap within 0.5 sec of the critical breath following an apnea/hypopnea, while the AASM 2007 extends this to exclude LM activity across entire respiratory events (i.e. 0.5 sec prior until 0.5 sec after respiratory events). Interestingly, however, we found these guidelines arbitrary and not substantiated by our data. Indeed, using this rule initially computed PLMS/h was greater in individuals with AHI>15, and lesser in those with AHI ≤15, suggesting false detections (see methods).

Examination of EMG activity reflecting LM time locked with respiratory events was thus next performed, and the 0.5 sec window applied to respiratory event boundaries inadequate to remove LM secondary to SDB, as EMG activity peaks approximately 3 sec following SDB (see Fig. 2). More surprisingly, we also found increased EMG a few seconds before the initiation of an SDB event. Whereas jerking activity of the body is commonly known to accompany the recovery breath at respiratory event offset, increased activity *prior* to respiratory event onset was not anticipated and several steps were taken to examine the results for error or explanation. For example, we only included isolated events without SDB immediately prior each event analyzed to avoid cofounding effects of prior events, but this did not change the signal. Not finding any artificial explanation, we conclude the finding to be physiological, perhaps reflecting a brief jerk when the airway is first obstructed or the leg jerk may precipitate a sharp inspiration sufficient to close or narrow a (pathologically) compliant airway. Alternatively, LM and SDB may be connected in a more complex way in some cases, for example via changes in arousal threshold that are known to be associated with LM or SDB generation [41], [42], [43]. Some investigators have suggested that patients with SDB may have increased occurrence of true PLMs independent of false LM detections [39]. It is also notable that PLM are associated with heart rate changes prior to the event [35], [44], and that K complexes and arousals associated with PLMs have been shown to occur periodically without the motor event, for example when patients with RLS are treated with dopamine agonists [35], [41], [42].

These results not withstanding, the new exclusion window proposed, 5 sec before initiation (plus 0.5 sec within the SDB event) and 5 sec after termination (plus 0.5 sec before termination), is based on empirical examination of leg EMG activity time locked to manually scored respiratory events that shows the strongest association at the ends (i.e. onset and offset) (see S6 and S7 Figures). Using this rule, PLMS/h did not differ between subjects with and without SDB (Table 4 and 5), indicating success of the algorithm for clinical purpose (i.e. when a physician simply wants to be sure PLMs are not only due to LM in association with abnormal breathing events). Interestingly however, in the WSC, PLMW was higher in subjects with AHI>15, possibly reflecting unscored breathing events during wakefulness, or just increased movements in patients with sleep apnea due to breathing discomfort when lying down. We also found that the periodicity index in sleep was significantly lower in subjects with AHI>15, a finding likely explained by the conservative exclusion rule around breathing events which will remove genuine PLMs that appear coincidently with breathing events. Additional descriptive research using the detector in patients with various degree and type of SDB may shed more light on this complex relation, so that better exclusion rules can be generated.

In the course of this study, we found that heart rate activation in association with PLMs was lower in subjects with SDB versus without. This preliminary result may reflect decreased cardiovascular response in these subjects or increased arousal threshold due to sleepiness [45]. Indeed, it is striking to note that most patients with SDB do not recall the severe sleep disruption associated with breathing events. Still there are several nuances with measuring cardiac activity with PLM that effect the outcome as presented by Ferri’s excellent commentary [35]. Further, heart rate activation with PLM varies across sleep stages, and this analysis was not performed [46]. Our goal here is not to provide definite explanation on the matter, but rather to illustrate how our detector may be used to further explore these and other avenues of research related to PLM.

In comparison with other detectors, our S-PLMAD detector fared best. Validation of the detector in two independent cohorts in comparison with gold standard, manual scoring using AASM 2007 criteria, revealed a very high correlation (Fig. 3). The fact the validation samples were enriched in complex associations such as unusual pathologies (REM behavior disorder, narcolepsy etc.) or SDB gives confidence that this detector should perform well in multiple clinical settings. We hope that dissemination of the detector will ensure widespread use and help comparative studies of PLMs across the world.

## Supporting Information

S1 Figure **Extended PLM detection algorithm flowchart.** The PLM detection algorithm consists of 10 steps, which are outlined sequentially in parts 1, 2, and 3.
(TIFF)

S2 Figure **Adaptive filtering of cardiac interference compared to conventional finite-impulse-response filtering.** Cardiac interference is adaptively cancelled from the leg EMG channel using a recursive least squares adaptive filter which continually updates its weights to minimize the least mean square difference between its output from filtering the correlated noise (i.e. the ECG channel) and the desired response (i.e. the leg EMG channel). The filter’s output is tuned to the correlated noise, which when subtracted from the desired response of signal and noise leaves the clean signal behind as the error, which is the signal less the correlated noise (i.e. the leg EMG without cardiac interference). The lower section shows three examples of leg EMG activity. The original data is shown at the top of each example. Under the original data is the rectified version followed by high pass filtered outputs with cutoff frequencies of 15 Hz, then 30 Hz, and finally the adaptively filtered data is shown at the bottom for each example. Horizontal bars show detections above the filtered data shown. The adaptively filtered data provides the best results in each case.
(TIFF)

S3 Figure **Examples of adaptive filtering to remove cardiac interference found in the leg EMG channel.** Panels A, B, C, D show the leg EMG channel on top, followed by the ECG channel second, the adaptive noise cancelled EMG channel using the ECG as input, and finally the adaptive noise cancelled EMG channel using a time shifted copy of the EMG as input (i.e. single channel configuration). The time shifted, self-reference adaptive filter configuration is less effective in cleaning the data than the ECG configuration, but still better than the original data. It is not disrupted by noise in the ECG channel as seen in B. For methodological details, see text.
(TIFF)

S4 Figure **Frequency response for SNR+2-tap summing filter.** A 2-tap FIR summing filter is used to raise the signal to noise ratio when the noise floor is small (i.e. less than 2 µV). The filter’s frequency response, using a 100 Hz sampling rate, is shown in panel A. The filter has linear phase delay (A bottom) and increases signal amplitude at frequencies below 33 Hz while further attenuating signal strength above 33 Hz - high frequency activity commonly attributed to noise. Detection algorithms that apply low or high pass filters using at 16 Hz cutoffs remove relevant portions of the surrounding spectral activity. Panel B shows the normalized magnitude response of the 2-tap filter in cascade with a simulated 10 Hz high pass hardware filter which is applied before digitization. Panels C, D, and E show the effect of applying the SNR+ option to one minute of leg EMG activity as a progression of steps. The top signal shows the input leg EMG activity (1), which is adaptively cleaned for ECG interference (2), smoothed via root mean square (3), and finally boosted by the SNR+ option (4).
(TIFF)

S5 Figure **Examples of a low noise floor.** In many cases, a low noise floor, defined as 2 µV or less, the leg EMG signal is attenuated and observed LM do not meet AASM amplitude criteria. In the study it was found that many LM marked for PLM were clearly visible when viewed on the one or two minute interval time scale commonly used by technicians. However, on close examination, these LM do not in fact meet the AASM 2007’s amplitude criteria. Three different time scales of stage 2 sleep are shown below: (A) 30 seconds, (B) 2 minutes, (C) 5 minutes. The raw input leg EMG channel is shown along the bottom of each view, with the adaptively filtered versions passed as input to the two detection configurations directly above. Detections made using the SNR+ option are shown as orange boxes along the top channel, while detections made without the SNR+ option are shown as magenta colored boxes above the middle channel.
(TIFF)

S6 Figure **Leg EMG activity time locked to manually scored WSC respiratory events.** Mean and median measures are taken of absolute leg EMG voltage at 0.5-second increments starting 30 s prior until 30 s after respiratory event onset (left figure) and each respiratory event offset (right figure). Leg EMG activity increases and then decreases prior to respiratory onset with a peak seen 5.5 s prior to onset. Leg EMG activity decreases and then increases at respiratory offset with a peak at 2.5 s (mean) or 3.5 s (median) following the exact point scored as offset. The pre-onset bump in EMG activity could be attributed to time locking respiratory events with short inter event intervals (e.g. a five second lapse between the end of one respiratory event and the start of the next) or short duration (e.g. less than 15 s). S7 and S8 Figures show the onset-to-onset and offset-to-onset interval distributions for successive respiratory events in the WSC.
(TIFF)

S7 Figure **Interval distributions for all manually scored WSC respiratory events shown in 5 s increments.** (a) Left plot is of onset to onset interval (b) Right plot is offset-to-onset interval (i.e. from the end of one respiratory event until the beginning of the next). The onset-to-onset interval peak occurs at 35 s, while the offset-to-onset interval peaks around 5-seconds. These distributions do not clarify whether the rise in EMG activity seen at time locked respiratory events is related to the offset of a respiratory event or the occurrence of the following respiratory event (which is often 5.0 to 10 s later in SDB).
(TIFF)

S8 Figure **Interval distribution of isolated respiratory events.** Manually scored respiratory events greater than 15 s duration and separated by more than 30 s from other respiratory events were identified to obtain the time locked leg EMG activity shown in Fig. 2, which in turn led to our proposed respiratory exclusion criteria. The distribution of the time between these respiratory events (i.e. the interval) is grouped in 5 s bins here. The left histogram shows their onset-to-onset interval distribution, while the right histogram shows their onset-to-offset interval distribution.
(TIFF)

S9 Figure **EMG activity time locked to respiratory events.** Per event EMG average in studies with only a single scored respiratory events (mean). Increased activity is still observed in this small patient sample, though the spike in activity following respiratory offset (right) is significantly higher than pre-onset activity (left).
(TIFF)

S10 Figure **Amplitude threshold functions of noise floor.** The upper threshold, *α*(*n*), increases as a function of the noise floor *η*(*n*), which is calculated for each sample point *n* of the leg EMG using a 20 s moving average filter. The lower threshold, *β*(*n*), is scaled using the ratio of the AASM 2007 Scoring Manuals upper and lower constant threshold values (i.e. 2 µV over 8 µV or 0.25). The cutoff threshold, *ψ*(*n*), is the average of the upper and lower thresholds and used to determine offset of candidate LMs. The detector shuts off whenever the noise floor exceeds 50 µV. Candidate LM detection onset occurs when the cleaned EMG signal first exceeds the upper threshold and terminates at the last point the cleaned EMG signal falls below the cutoff threshold prior to subsequently falling below the lower threshold for 0.05 s.
(TIFF)

S1 Table **Previously published PLM detectors.**
(DOC)

S2 Table **Preliminary detector performance.** Preliminary detector performance compared to manually scored LM in the WSC, during sleep, according to WSC 1995 criteria (see text).
(DOC)

S3 Table **PLM detector comparisons.** Detector performance compared to manually scored PLM in the WSC (upper table, n = 60 subjects) and the SSC (lower table, n = 18 subjects), during sleep according to AASM 2007 criteria. Tauchmann, Ferri, and Wetter detectors were only evaluated in the WSC, during the development stage.
(DOC)

S4 Table **Impact of different respiratory exclusion rules.** Several respiratory exclusion rules are applied to the PLM detector (adaptive filtering) to evaluate and compare their effect on median LM count, and mean and median PLMI in patients with AHI ≥15 and with AHI <15 during sleep. The AASM 2007 exclusion window (indicated by *) reveals significantly higher median LM count and PLMS/h with increased AHI. Pathological PLMs should be similar in both groups, and onset and offset respiratory exclusion criteria are optimized to this end here. LM count shows the fluctuations caused by different windows prior to application of PLM criteria. Several suitable exclusion choices exist for removing activity associated with respiratory event that produce equal PLM detections in patients with and without SDB. We selected −5.0 to 0.5 around onset and −0.5 to 5.0 s around offset because of its good performance and relative symmetry.
(DOC)
