# Correction: Design and Validation of a Periodic Leg Movement Detector

**DOI:** 10.1371/journal.pone.0138205

**Published:** 2015-09-10

**Authors:** Hyatt Moore, Eileen Leary, Seo-Young Lee, Oscar Carrillo, Robin Stubbs, Paul Peppard, Terry Young, Bernard Widrow, Emmanuel Mignot

The affiliation for the third author is incomplete. Seo-Young Lee is also affiliated with: Kangwon National University School of Medicine, Chuncheon, South Korea.
