# Supplementary figures and images for: Design and Validation of a Periodic Leg Movement Detector
Source: PLoS One. 2014 Dec 9;9(12):e114565. doi: 10.1371/journal.pone.0114565 (PMC4260847; doi:10.1371/journal.pone.0114565)

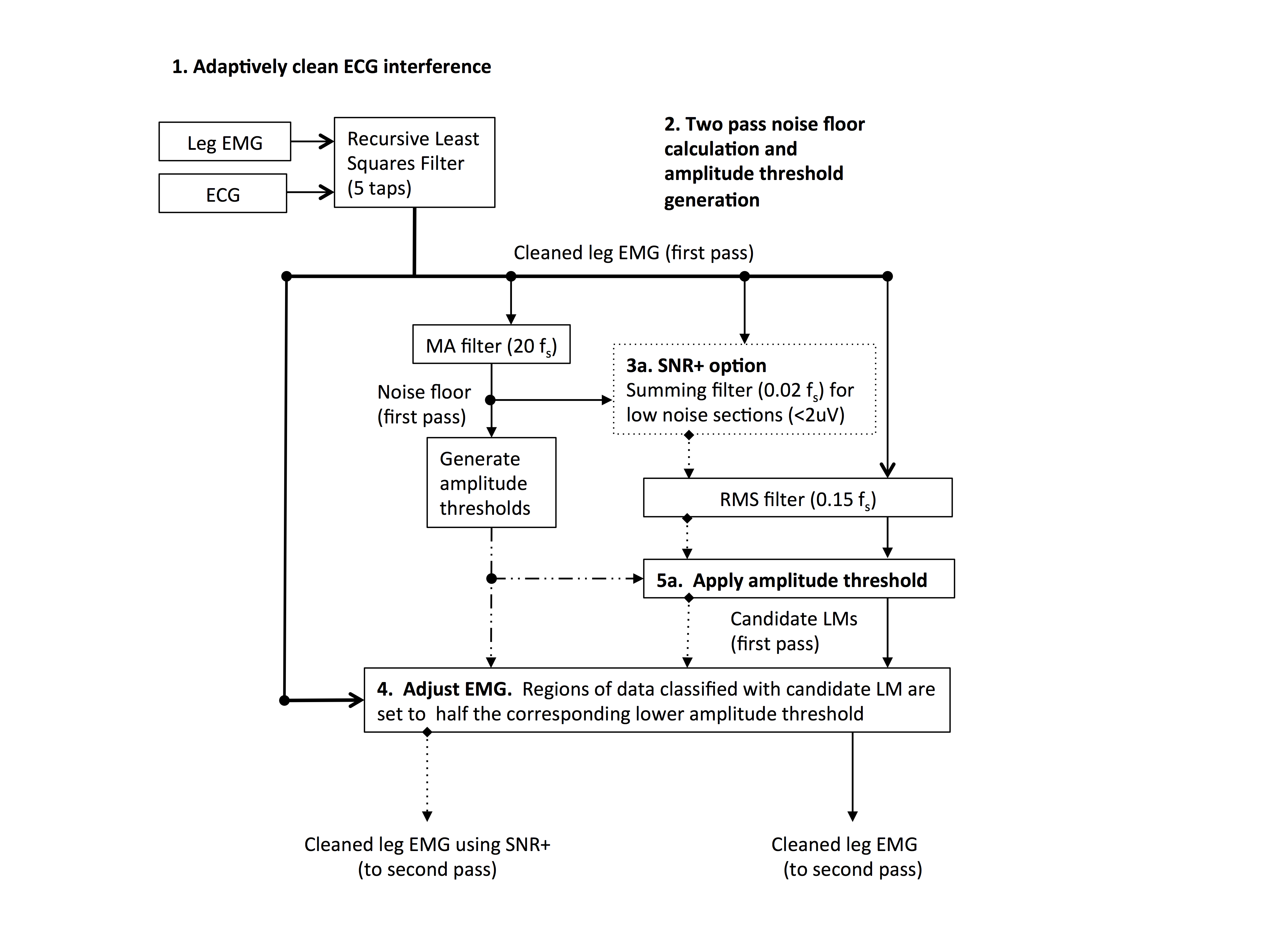

Supplement: S1 Figure — Extended PLM detection algorithm flowchart. The PLM detection algorithm consists of 10 steps, which are outlined sequentially in parts 1, 2, and 3. (TIFF) [file pone.0114565.s001.tiff]

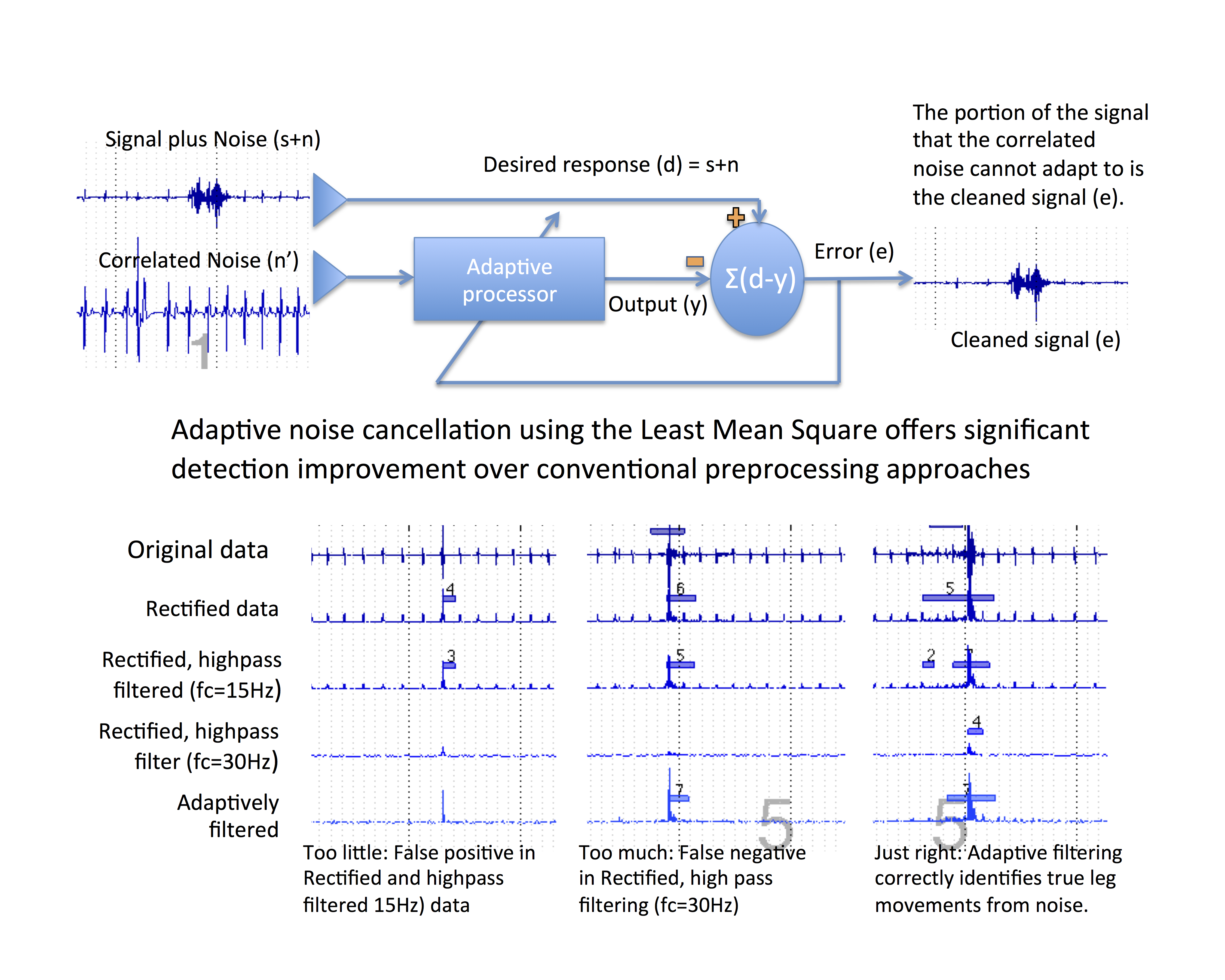

Supplement: S2 Figure — Adaptive filtering of cardiac interference compared to conventional finite-impulse-response filtering. Cardiac interference is adaptively cancelled from the leg EMG channel using a recursive least squares adaptive filter which continually updates its weights to minimize the least mean square difference between its output from filtering the correlated noise (i.e. the ECG channel) and the desired response (i.e. the leg EMG channel). The filter’s output is tuned to the correlated noise, which when subtracted from the desired response of signal and noise leaves the clean signal behind as the error, which is the signal less the correlated noise (i.e. the leg EMG without cardiac interference). The lower section shows three examples of leg EMG activity. The original data is shown at the top of each example. Under the original data is the rectified version followed by high pass filtered outputs with cutoff frequencies of 15 Hz, then 30 Hz, and finally the adaptively filtered data is shown at the bottom for each example. Horizontal bars show detections above the filtered data shown. The adaptively filtered data provides the best results in each case. (TIFF) [file pone.0114565.s002.tiff]

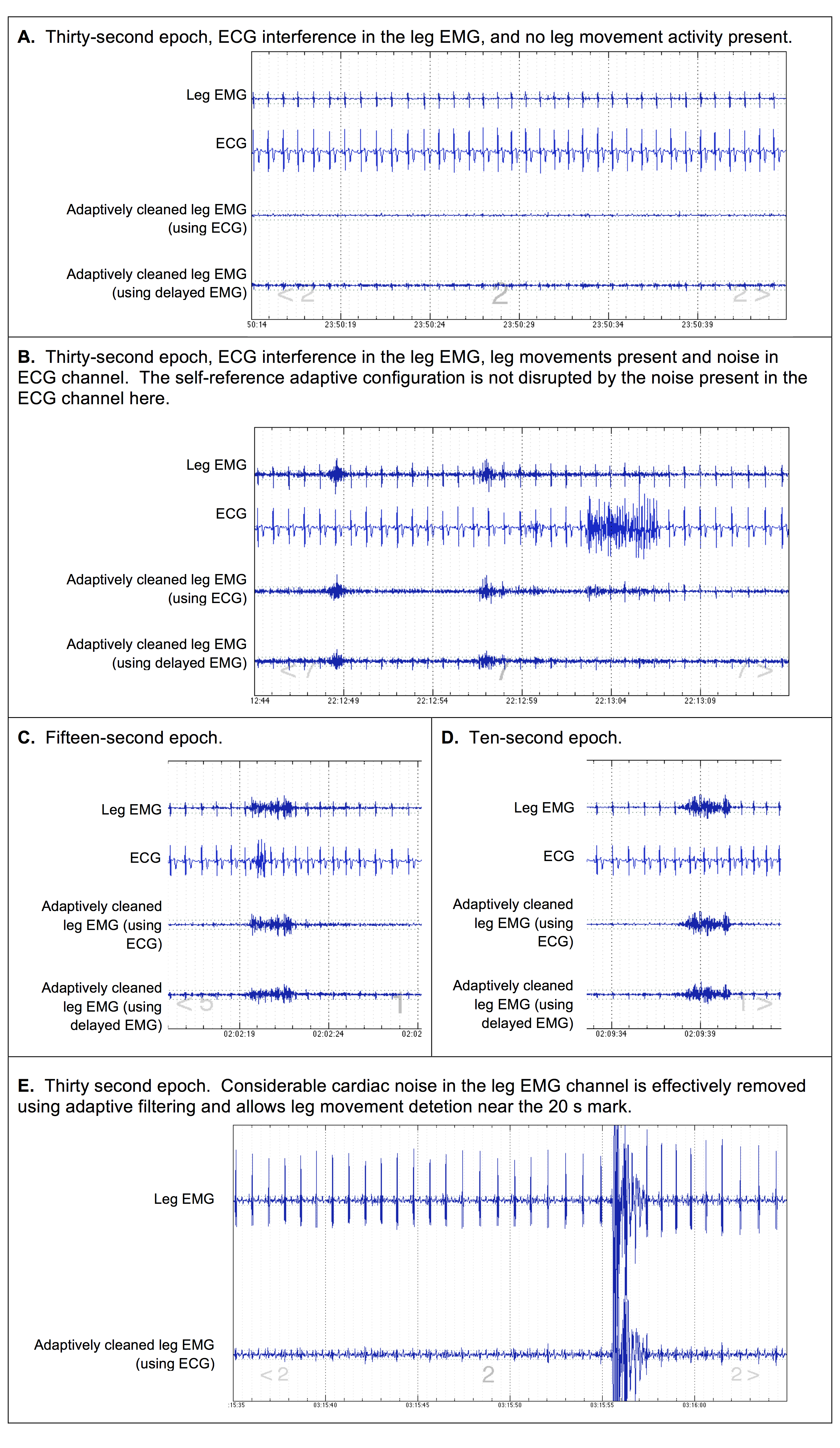

Supplement: S3 Figure — Examples of adaptive filtering to remove cardiac interference found in the leg EMG channel. Panels A, B, C, D show the leg EMG channel on top, followed by the ECG channel second, the adaptive noise cancelled EMG channel using the ECG as input, and finally the adaptive noise cancelled EMG channel using a time shifted copy of the EMG as input (i.e. single channel configuration). The time shifted, self-reference adaptive filter configuration is less effective in cleaning the data than the ECG configuration, but still better than the original data. It is not disrupted by noise in the ECG channel as seen in B. For methodological details, see text. (TIFF) [file pone.0114565.s003.tiff]

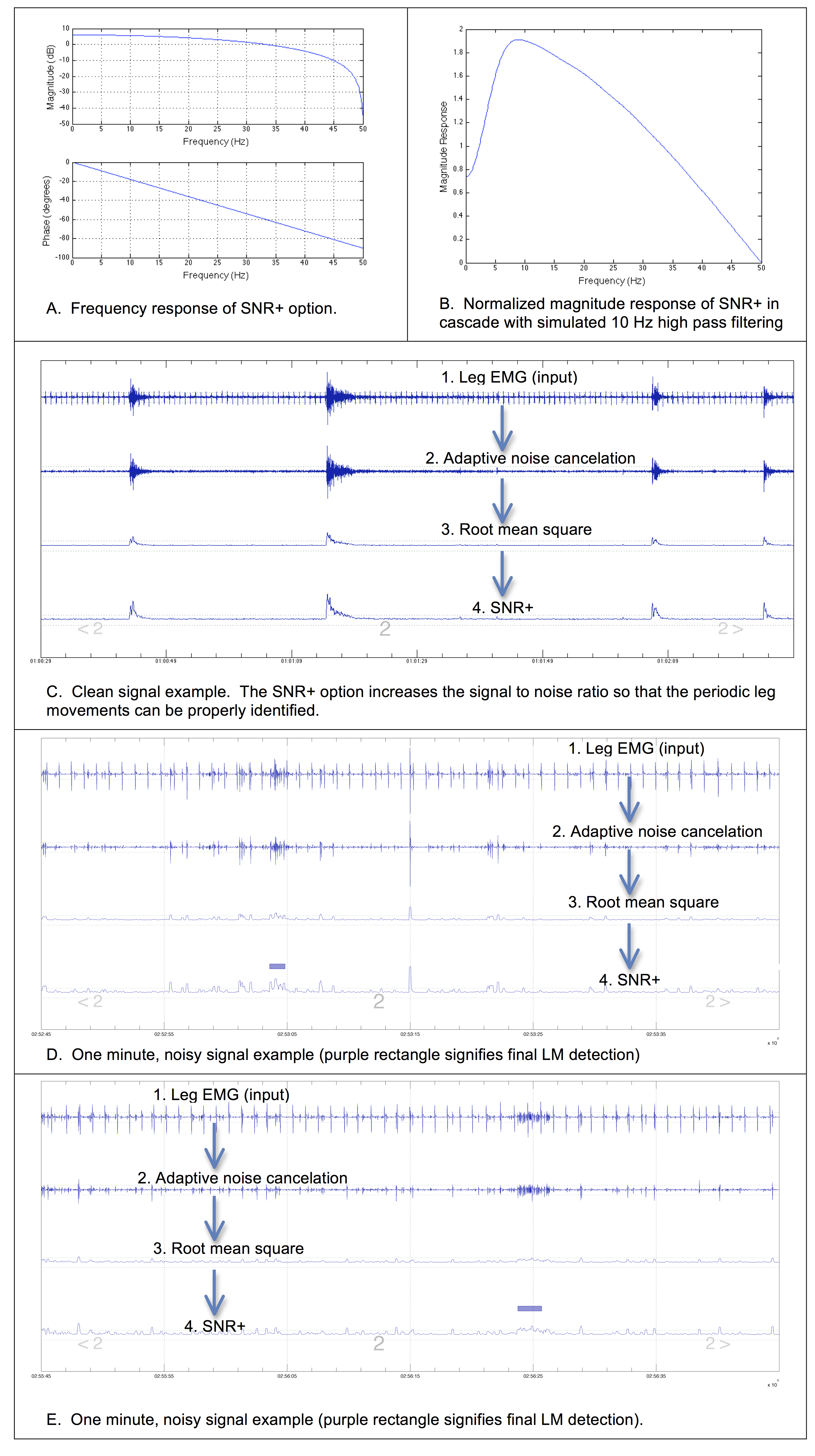

Supplement: S4 Figure — Frequency response for SNR+2-tap summing filter. A 2-tap FIR summing filter is used to raise the signal to noise ratio when the noise floor is small (i.e. less than 2 µV). The filter’s frequency response, using a 100 Hz sampling rate, is shown in panel A. The filter has linear phase delay (A bottom) and increases signal amplitude at frequencies below 33 Hz while further attenuating signal strength above 33 Hz - high frequency activity commonly attributed to noise. Detection algorithms that apply low or high pass filters using at 16 Hz cutoffs remove relevant portions of the surrounding spectral activity. Panel B shows the normalized magnitude response of the 2-tap filter in cascade with a simulated 10 Hz high pass hardware filter which is applied before digitization. Panels C, D, and E show the effect of applying the SNR+ option to one minute of leg EMG activity as a progression of steps. The top signal shows the input leg EMG activity (1), which is adaptively cleaned for ECG interference (2), smoothed via root mean square (3), and finally boosted by the SNR+ option (4). (TIFF) [file pone.0114565.s004.tiff]

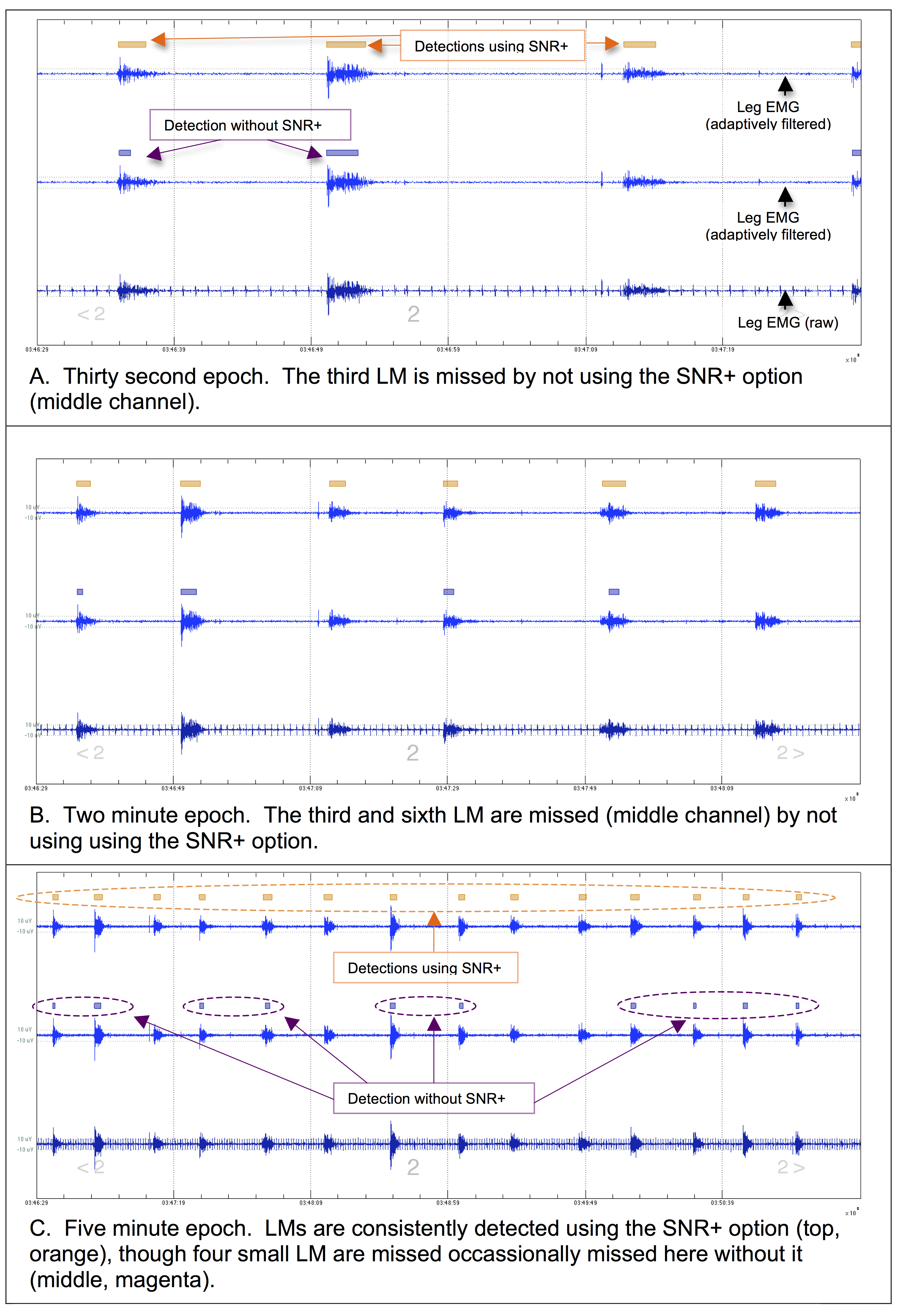

Supplement: S5 Figure — Examples of a low noise floor. In many cases, a low noise floor, defined as 2 µV or less, the leg EMG signal is attenuated and observed LM do not meet AASM amplitude criteria. In the study it was found that many LM marked for PLM were clearly visible when viewed on the one or two minute interval time scale commonly used by technicians. However, on close examination, these LM do not in fact meet the AASM 2007’s amplitude criteria. Three different time scales of stage 2 sleep are shown below: (A) 30 seconds, (B) 2 minutes, (C) 5 minutes. The raw input leg EMG channel is shown along the bottom of each view, with the adaptively filtered versions passed as input to the two detection configurations directly above. Detections made using the SNR+ option are shown as orange boxes along the top channel, while detections made without the SNR+ option are shown as magenta colored boxes above the middle channel. (TIFF) [file pone.0114565.s005.tiff]

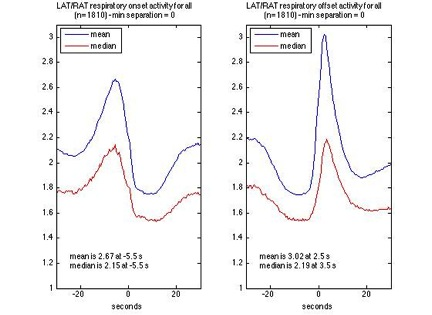

Supplement: S6 Figure — Leg EMG activity time locked to manually scored WSC respiratory events. Mean and median measures are taken of absolute leg EMG voltage at 0.5-second increments starting 30 s prior until 30 s after respiratory event onset (left figure) and each respiratory event offset (right figure). Leg EMG activity increases and then decreases prior to respiratory onset with a peak seen 5.5 s prior to onset. Leg EMG activity decreases and then increases at respiratory offset with a peak at 2.5 s (mean) or 3.5 s (median) following the exact point scored as offset. The pre-onset bump in EMG activity could be attributed to time locking respiratory events with short inter event intervals (e.g. a five second lapse between the end of one respiratory event and the start of the next) or short duration (e.g. less than 15 s). S7 and S8 Figures show the onset-to-onset and offset-to-onset interval distributions for successive respiratory events in the WSC. (TIFF) [file pone.0114565.s006.tiff]

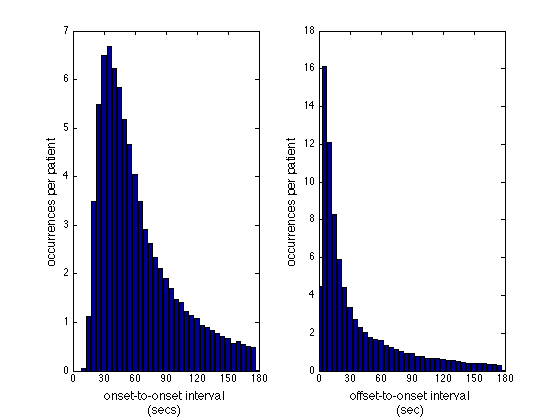

Supplement: S7 Figure — Interval distributions for all manually scored WSC respiratory events shown in 5 s increments. (a) Left plot is of onset to onset interval (b) Right plot is offset-to-onset interval (i.e. from the end of one respiratory event until the beginning of the next). The onset-to-onset interval peak occurs at 35 s, while the offset-to-onset interval peaks around 5-seconds. These distributions do not clarify whether the rise in EMG activity seen at time locked respiratory events is related to the offset of a respiratory event or the occurrence of the following respiratory event (which is often 5.0 to 10 s later in SDB). (TIFF) [file pone.0114565.s007.tiff]

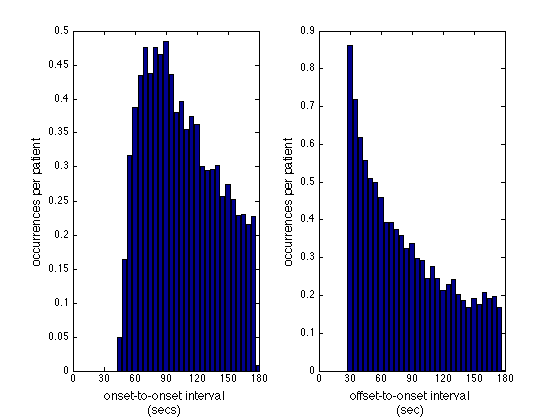

Supplement: S8 Figure — Interval distribution of isolated respiratory events. Manually scored respiratory events greater than 15 s duration and separated by more than 30 s from other respiratory events were identified to obtain the time locked leg EMG activity shown in Fig. 2, which in turn led to our proposed respiratory exclusion criteria. The distribution of the time between these respiratory events (i.e. the interval) is grouped in 5 s bins here. The left histogram shows their onset-to-onset interval distribution, while the right histogram shows their onset-to-offset interval distribution. (TIFF) [file pone.0114565.s008.tiff]

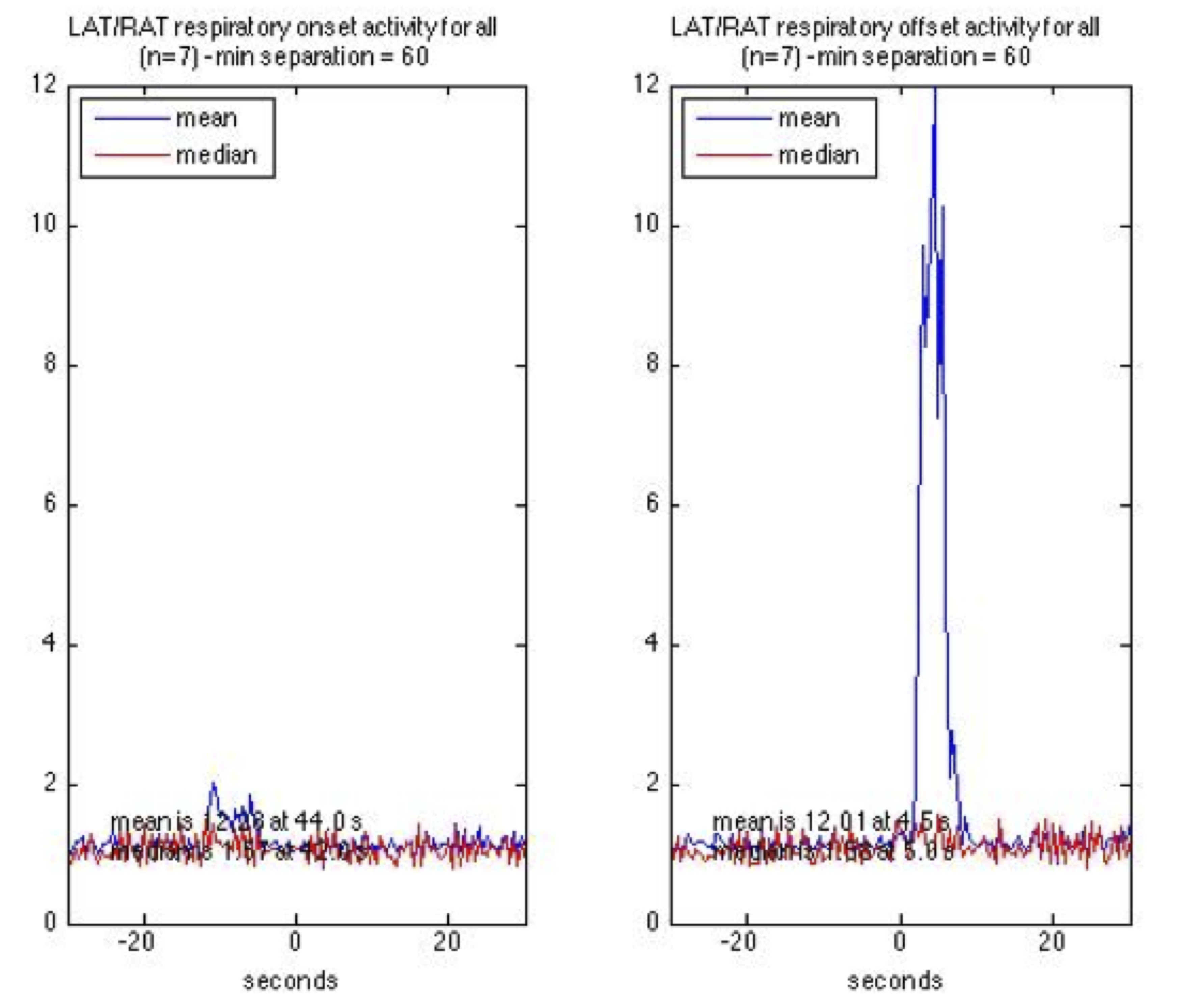

Supplement: S9 Figure — EMG activity time locked to respiratory events. Per event EMG average in studies with only a single scored respiratory events (mean). Increased activity is still observed in this small patient sample, though the spike in activity following respiratory offset (right) is significantly higher than pre-onset activity (left). (TIFF) [file pone.0114565.s009.tiff]

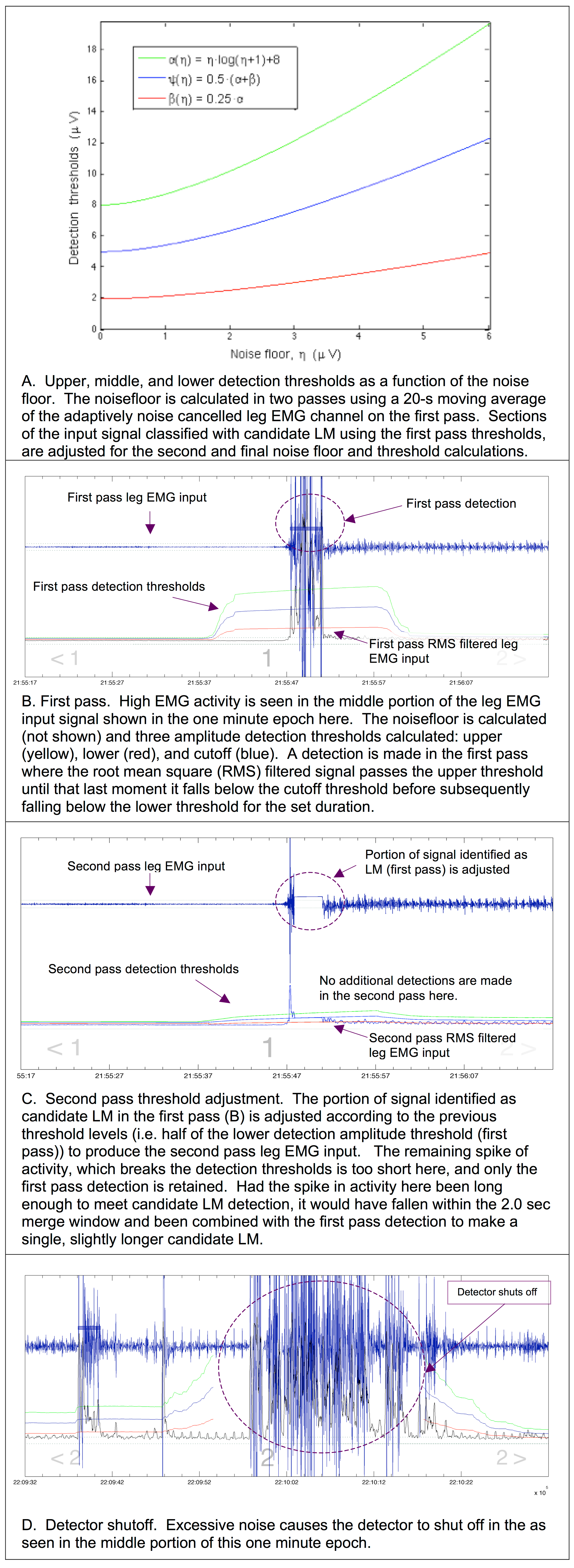

Supplement: S10 Figure — Amplitude threshold functions of noise floor. The upper threshold, α(n), increases as a function of the noise floor η(n), which is calculated for each sample point n of the leg EMG using a 20 s moving average filter. The lower threshold, β(n), is scaled using the ratio of the AASM 2007 Scoring Manuals upper and lower constant threshold values (i.e. 2 µV over 8 µV or 0.25). The cutoff threshold, ψ(n), is the average of the upper and lower thresholds and used to determine offset of candidate LMs. The detector shuts off whenever the noise floor exceeds 50 µV. Candidate LM detection onset occurs when the cleaned EMG signal first exceeds the upper threshold and terminates at the last point the cleaned EMG signal falls below the cutoff threshold prior to subsequently falling below the lower threshold for 0.05 s. (TIFF) [file pone.0114565.s010.tiff]
