# Supplementary material for: Design and Validation of a Periodic Leg Movement Detector
Source: PLoS One. 2014 Dec 9;9(12):e114565. doi: 10.1371/journal.pone.0114565 (PMC4260847; doi:10.1371/journal.pone.0114565)
Supplement: S1 Table — Previously published PLM detectors. (DOC) [file pone.0114565.s011.doc]

**Table S1. Characteristics of previously published PLM detectors.**

|  | Classification rules | Preprocessing | Postprocessing |
| --- | --- | --- | --- |
| All | Duration between 0.5 and 10.0 s  (Amplitude criteria in reference to baseline) | Notch filtering for power line interference |  |
| Tauchmann (1996) | Onset-offset amplitude > 7 µV  Arithmetic mean > 5 µV | Low pass filter (f_c_=16Hz)  Rectify | Merge consecutive activity within 0.15 sec |
| Wetter (2004) | Continuous burst activity (standard deviation of amplitude>0.6 µV) for 0.4 s of 0.5 s segments. | High pass filter (f_c_=16Hz)  Rectify  Truncate to 30 µV  16 ms standard deviation window | Merge consecutive activity within 0.5 sec |
| Ferri (2005) | Onset amplitude > 7 µV  Offset amplitude < 2 µV | High pass filter (f_c_=16Hz)  0.5 s moving average filter for offset amplitude |  |

Tauchmann NPT. Automatic Detection of Periodic Leg Movements. J Sleep Res 1996;5(4):273-5.

Wetter TC, Dirlich G, Streit J, Trenkwalder C, Schuld A, Pollmacher T. An automatic method for scoring leg movements in polygraphic sleep recordings and its validity in comparison to visual scoring. Sleep 2004;27(2):324-8.

Ferri R, Zucconi M, Manconi M, et al. Computer-assisted detection of nocturnal leg motor activity in patients with restless legs syndrome and periodic leg movements during sleep. Sleep 2005;28(8):998-1004.
