# Supplementary material for: Design and Validation of a Periodic Leg Movement Detector
Source: PLoS One. 2014 Dec 9;9(12):e114565. doi: 10.1371/journal.pone.0114565 (PMC4260847; doi:10.1371/journal.pone.0114565)
Supplement: S2 Table — Preliminary detector performance. Preliminary detector performance compared to manually scored LM in the WSC, during sleep, according to WSC 1995 criteria (see text). (DOC) [file pone.0114565.s012.doc]

**Table S2. Preliminary detector performance. Preliminary detector performance compared to manually scored LM in the WSC, during sleep, according to WSC 1995 criteria*.**

|  | SE | | SP | | PPV | | NPV | | Cohen's Kappa | | ACC | | | LM  Count | | |  |
| --- | --- | --- | --- | --- | --- | --- | --- | --- | --- | --- | --- | --- | --- | --- | --- | --- | --- |
| **Detector performance**  **for all PLM in WSC** | |  | |  | |  | |  | |  | |  | | |  | | |
| Tauchmann | 0.30 | | 0.98 | | 0.21 | | 0.99 | | 0.24 | | 0.97 | | | 167,955 | | |  |
| Wetter | 0.86 | | 0.95 | | 0.20 | | 1.00 | | 0.31 | | 0.95 | | | 503,211 | | |  |
| Ferri | 0.94 | | 0.87 | | 0.09 | | 1.00 | | 0.14 | | 0.87 | | | 1,220,765 | | |  |
| SNR+ | 0.78 | | 1.00 | | 0.73 | | 1.00 | | 0.75 | | 0.99 | | | 124,351 | | |  |
| ANC, SNR+ | 0.75 | | 1.00 | | 0.74 | | 1.00 | | 0.74 | | 0.99 | | | 116,335 | | |  |
| ANC, VAT | 0.53 | | 1.00 | | 0.80 | | 0.99 | | 0.64 | | 0.99 | | | 76,585 | | |  |
| ANC, VAT, SNR+ | 0.72 | | 1.00 | | 0.79 | | 1.00 | | 0.75 | | 0.99 | | | 106,397 | | |  |
| *WSC visually scored* | 1.00 | | 1.00 | | 1.00 | | 1.00 | | 1.00 | | 1.00 | | | 119,277 | | |  |
| **Detector performance**  **for all PLM in SSC** | | | | | | | | | | | | |  | | |  |  |
| Tauchmann | 0.26 | | 0.98 | | 0.37 | | 0.99 | | 0.25 | | 0.97 | | | 156.53 | | |  |
| Wetter | 0.85 | | 0.95 | | 0.30 | | 1.00 | | 0.39 | | 0.95 | | | 468.98 | | |  |
| Ferri | 0.93 | | 0.89 | | 0.31 | | 0.96 | | 0.42 | | 0.89 | | | 1,137.71 | | |  |
| SNR+ | 0.75 | | 1.00 | | 0.69 | | 1.00 | | 0.69 | | 0.99 | | | 115.89 | | |  |
| SNR+, ANC | 0.71 | | 1.00 | | 0.71 | | 1.00 | | 0.67 | | 0.99 | | | 108.42 | | |  |
| ANC, VAT | 0.44 | | 1.00 | | 0.68 | | 0.99 | | 0.50 | | 0.99 | | | 71.37 | | |  |
| SNR+, ANC, VAT | 0.65 | | 1.00 | | 0.69 | | 1.00 | | 0.64 | | 0.99 | | | 99.16 | | |  |
| *SSC visually scored* | 1.00 | | 1.00 | | 1.00 | | 1.00 | | 1.00 | | 1.00 | | | 111.16 | | |  |

*See text for criteria description. ANC: adaptive noise cancelling of cardiac interference; SNR+: signal-to-noise-ratio enhancement; VAT: Variable amplitude thresholding; SE: sensitivity; SP: specificity; PPV: Positive predictive value; NPV: negative predictive value; ACC: accuracy; LM count: total number of leg movements detected.
