# Supplementary material for: Design and Validation of a Periodic Leg Movement Detector
Source: PLoS One. 2014 Dec 9;9(12):e114565. doi: 10.1371/journal.pone.0114565 (PMC4260847; doi:10.1371/journal.pone.0114565)
Supplement: S3 Table — PLM detector comparisons. Detector performance compared to manually scored PLM in the WSC (upper table, n = 60 subjects) and the SSC (lower table, n = 18 subjects), during sleep according to AASM 2007 criteria. Tauchmann, Ferri, and Wetter detectors were only evaluated in the WSC, during the development stage. (DOC) [file pone.0114565.s013.doc]

**Table S3. PLM detector comparisons. Detector performance compared to manually scored PLM in the WSC (upper table, n=60 subjects) and the SSC (lower table, n=18 subjects), during sleep according to AASM 2007 criteria. Tauchmann, Ferri, and Wetter detectors were only evaluated in the WSC, during the development stage.**

|  | SE | SP | | PPV | NPV | Cohen's Kappa | ACC | PLM  Count |
| --- | --- | --- | --- | --- | --- | --- | --- | --- |
| **Detector performance for all PLM in WSC** | |  | |  |  |  |  |  |
| Tauchmann | 0.24 | 1.00 | | 0.79 | 0.97 | 0.36 | 0.97 | 1,436 |
| Wetter | 0.96 | 0.98 | | 0.47 | 1.00 | 0.62 | 0.98 | 11,160 |
| Ferri | 0.85 | 0.99 | | 0.62 | 1.00 | 0.72 | 0.99 | 7,237 |
| ANC, VAT | 0.60 | 1.00 | | 0.88 | 0.99 | 0.71 | 0.98 | 3,443 |
| ANC, VAT, SNR+ | 0.73 | 1.00 | | 0.85 | 0.99 | 0.78 | 0.99 | 4,517 |
| *WSC visually scored* | 1.00 | 1.00 | | 1.00 | 1.00 | 1.00 | 1.00 | 5,434 |
| **Detector performance for all PLM in SSC** |  |  |  | |  |  |  |  |
| ANC, VAT | 0.75 | 1.00 | 0.82 | | 0.99 | 0.78 | 0.99 | 1,591 |
| ANC, VAT, SNR+ | 0.77 | 0.99 | 0.77 | | 0.99 | 0.76 | 0.99 | 1,732 |
| *SSC visually scored* | 1.00 | 1.00 | 1.00 | | 1.00 | 1.00 | 1.00 | 1,733 |

ANC: adaptive noise cancelling of cardiac interference; SNR+: signal-to-noise ratio enhancement; VAT: Variable amplitude thresholding; SE: sensitivity; SP: specificity; PPV: Positive predictive value; NPV: negative predictive value; ACC: accuracy; PLM count: total number of periodic leg movements detected.
