# Supplementary material for: Design and Validation of a Periodic Leg Movement Detector
Source: PLoS One. 2014 Dec 9;9(12):e114565. doi: 10.1371/journal.pone.0114565 (PMC4260847; doi:10.1371/journal.pone.0114565)
Supplement: S4 Table — Impact of different respiratory exclusion rules. Several respiratory exclusion rules are applied to the PLM detector (adaptive filtering) to evaluate and compare their effect on median LM count, and mean and median PLMI in patients with AHI ≥15 and with AHI <15 during sleep. The AASM 2007 exclusion window (indicated by *) reveals significantly higher median LM count and PLMS/h with increased AHI. Pathological PLMs should be similar in both groups, and onset and offset respiratory exclusion criteria are optimized to this end here. LM count shows the fluctuations caused by different windows prior to application of PLM criteria. Several suitable exclusion choices exist for removing activity associated with respiratory event that produce equal PLM detections in patients with and without SDB. We selected −5.0 to 0.5 around onset and −0.5 to 5.0 s around offset because of its good performance and relative symmetry. (DOC) [file pone.0114565.s014.doc]

**Table S4. Impact of different respiratory exclusion rules.** Several respiratory exclusion rules are applied to the PLM detector (adaptive filtering) to evaluate and compare their effect on median LM count, and mean and median PLMI in patients with AHI ≥ 15 and with AHI < 15 during sleep. The AASM 2007 exclusion window (indicated by *) reveals significantly higher median LM count and PLMS/h with increased AHI. Pathological PLMs should be similar in both groups, and onset and offset respiratory exclusion criteria are optimized to this end here. LM count shows the fluctuations caused by different windows prior to application of PLM criteria. Several suitable exclusion choices exist for removing activity associated with respiratory event that produce equal PLM detections in patients with and without SDB. We selected -5.0 to 0.5 around onset and -0.5 to 5.0 s around offset because of its good performance and relative symmetry.

| Onset | Offset | Median LM count | | Median PLMS/h | | Mean PLMS/h | |
| --- | --- | --- | --- | --- | --- | --- | --- |
| (before, after) | (before, after) | AHI<15 | AHI≥15 | AHI<15 | AHI≥15 | AHI<15 | AHI≥15 |
| None | None | 84 | 154 | 4.8 | 12.6 | 14.1 | 23.6 |
| (-0.5, ~)* | (~, 0.5)* | 73 | 107 | 3.43 | 5.9 | 12.1 | 12.7 |
| (-10, 0.5) | (-0.5, 5.0) | 70 | 63 | 3.3 | 2.6 | 12.0 | 9.9 |
| (-10, 0.0) | (-0.5, 3.5) | 71 | 66 | 3.3 | 2.7 | 12.0 | 10 |
| (-5.0, 0.0) | (-0.5, 3.5) | 76 | 82 | 3.4 | 3.8 | 12.5 | 11.5 |
| (-7.5, 0.0) | (-0.5, 3.5) | 74 | 72 | 3.3 | 3.2 | 12.4 | 10.7 |
| **(-5.0, 0.5)** | **(-0.5, 5.0)** | **74** | **76** | **3.34** | **3.37** | **12.5** | **11.9** |
| (-5.0, 0.0) | (-0.5, 5.0) | 74 | 77 | 3.3 | 3.3 | 12.4 | 11.2 |
| (-5.0, 0.0) | (0.0, 5.0) | 74 | 79 | 3.3 | 3.6 | 12.5 | 11.3 |
| (-3.5, 0.0) | (-0.5, 3.5) | 76 | 91 | 3.5 | 4.4 | 12.6 | 12.0 |
| (-6.0, 0.0) | (-0.5, 3.5) | 75 | 77 | 3.4 | 3.8 | 12.5 | 11.1 |
| (-6.0, 0.5) | (-0.5, 3.5) | 75 | 77 | 3.4 | 3.8 | 12.4 | 11.1 |
| (-6.0, 1.0) | (-1.0, 3.5) | 74 | 74 | 3.4 | 3.6 | 12.4 | 10.9 |
| (-6.0, 1.0) | (-1.0, 4.0) | 73 | 73 | 3.3 | 3.3 | 12.4 | 10.8 |
| (-6.0, 1.0) | (-1.0, 5.0) | 72 | 72 | 3.3 | 3.2 | 12.3 | 10.6 |
| (-5.0, 1.0) | (-1.0, 5.0) | 73 | 75 | 3.3 | 3.2 | 12.4 | 10.9 |
| (-5.0, 1.0) | (-1.0, 6.0) | 72 | 72 | 3.3 | 3.1 | 12.4 | 10.7 |
| (-5.0, 1.0) | (-1.0, 7.5) | 72 | 69 | 3.3 | 2.8 | 12.3 | 10.4 |
| (-5.0, 5.0) | (-5.0, 5.0) | 71 | 70 | 3.3 | 2.8 | 12.1 | 9.9 |
| (-6.0, 5.0) | (-5.0, 4.0) | 71 | 68 | 3.3 | 2.9 | 12.1 | 9.8 |
| (-6.0, 2.0) | (-2.0, 4.0) | 72 | 72 | 3.3 | 3.2 | 12.2 | 10.5 |
| (-6.0, 2.0) | (-1.0, 5.0) | 79 | 102 | 4.0 | 4.7 | 13.0 | 13.6 |
| (-6.0, 2.0) | (-0.5, 5.0) | 79 | 102 | 4.0 | 5.1 | 13.0 | 13.8 |
| (-6.0, 2.0) | (-1.0, 7.5) | 77 | 93 | 3.9 | 4.0 | 12.9 | 12.6 |
| (-6.0, 2.0) | (-2.0, 7.5) | 78 | 92 | 3.9 | 3.9 | 12.8 | 12.4 |
| (-6.0, 1.0) | **(-**2.0, 7.5) | 79 | 100 | 3.9 | 5.8 | 13.1 | 14.9 |
| (-7.5, 1.0) | (-2.0, 7.5) | 79 | 100 | 3.9 | 5.8 | 13.1 | 14.9 |
| (-10.0, 1.0) | (-2.0, 7.5) | 77 | 87 | 3.8 | 4.2 | 12.8 | 13.1 |

*AASM 2007 respiratory exclusion criteria (i.e. 0.5 s prior through 0.5 s following a respiratory event.
